# Supplementary material for: Direct control of CAR T cells through small molecule-regulated antibodies
Source: Nat Commun. 2021 Jan 29;12:710. doi: 10.1038/s41467-020-20671-6 (PMC7846603; doi:10.1038/s41467-020-20671-6)
Supplement: Supplementary file 1 — Supplementary Information [file 41467_2020_20671_MOESM1_ESM.pdf]

Supplementary Figures

Supplementary Figure 1. Engineering and humanization of V_{HH} into an scFv.

a, Sequence alignment of VHH-MTX, M2J1, and the engineered conditional scaffold, from which M2J1-MTX is derived. Grey indicates regions critical for MTX binding. Blue indicates regions involved in VH:VL interface. Yellow indicates heavy chain CDRs. Red font indicates residues unique to anti-hCMV M2J1. b, Synthetic antibody library design. A scFv library with a predisposition to MTX binding was generated by individually pairing a humanized camelid V_{HH} (blue) with three different human antibody light chain germlines (V_k1-39 (red), V_k3-20 (yellow) and V_l1-47 (purple)). Heavy chain CDR1 and CDR2 were fixed to predispose binding toward MTX. Diversity was introduced around the remaining heavy and light chain CDRs at positions determined to interact with antigens based on crystal structure analysis and with an abundance of natural amino acid variability (green X's) to obtain a secondary specificity toward additional antigens. The diversity introduced was based on the natural amino acid distribution of non-redundant naïve B cell sequences from the immune repertoire of 228 donors[1]. Critical residues for MTX binding are underlined.

a

KABAT NUMBERING, VH ALIGNMENT

																										CDRH1								
	1	2	3	4	5	6	7	8	9	10	11	12	13	14	15	16	17	18	19	20	21	22	23	24	25	26	27	28	29	30	31	32	33	34
anti-MTX VHH Camelid	Q	V	Q	L	V	E	S	G	G	G	L	V	Q	A	G	G	S	L	R	L	S	C	A	A	S	R	R	S	S	R	S	W	A	M
anti-hCMV M2J1	-	-	-	-	-	-	-	-	-	-	V	-	-	P	-	R	-	-	-	-	-	-	-	-	-	G	F	T	F	S	-	Y	G	-
Conditional Scaffold	Q	V	Q	L	V	E	S	G	G	G	L	V	Q	A	G	G	S	L	R	L	S	C	A	A	S	R	R	S	S	R	S	W	A	M

	VH:VL									VH:VL								CDRH2							VH:VL									
	35	36	37	38	39	40	41	42	43	44	45	46	47	48	49	50	51	52	53	54	55	56	56A	57	58	59	60	61	62	63	64	65	66	67
anti-MTX VHH Camelid	A	W	F	R	Q	A	P	G	K	E	R	E	F	V	A	K	I	S	G	D	G	R	L	T	T	Y	G	D	S	V	K	G	R	F
anti-hCMV M2J1	H	-	V	-	-	-	-	-	-	G	L	-	W	-	-	V	-	-	Y	-	-	S	N	K	Y	-	A	-	-	-	-	-	-	-
Conditional Scaffold	H	W	V	R	Q	A	P	G	K	G	L	E	W	V	A	V	I	S	Y	D	G	R	L	K	Y	Y	A	D	S	V	K	G	R	F

						FW3																						CAA MOTIF			CDRH3
	68	69	70	71	72	73	74	75	76	77	78	79	80	81	82	82A	82B	82C	83	84	85	86	87	88	89	90	91	92	93	94	95
anti-MTX VHH Camelid	T	I	S	R	D	N	A	E	Y	L	V	Y	L	Q	M	D	S	L	K	P	E	D	T	A	V	Y	Y	C	A	A	D
anti-hCMV M2J1	-	-	-	-	-	-	S	K	N	T	L	-	-	-	-	N	-	-	R	A	-	-	-	-	-	-	-	-	-	K	-
Conditional Scaffold	T	I	S	R	D	N	A	E	Y	L	V	Y	L	Q	M	N	S	L	R	A	E	D	T	A	V	Y	Y	C	A	A	D

b

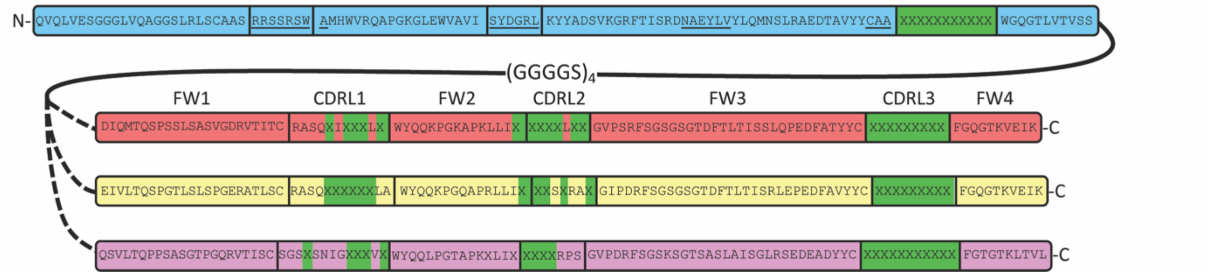

Supplementary Figure 2. Determining regions of CDRH3 critical for MTX binding.

Surface plasmon resonance was used to investigate the interaction of MTX with M2J1-scFv-Fc variants, where the CAA motif at the N-terminus of CDR H3 was mutated to CAS, CAN, CAT and CAR. Biotinlyated scFv-Fc-fusion proteins were captured on a streptavidin sensor chip and MTX was flowed as the analyte at 0.08 µM, 0.4 µM, 2 µM, 10 µM and 50 µM at 37°C. The figures on the left side show the sensorgrams for all M2J1 variants and steady-state affinity curve fits are shown on the right side. The sensorgrams for M2J1-scFv-Fc (CAA) were fit to a 1:1 Langmuir with mass transport kinetics model and the affinity was determined by the ratio of the rate constants. Kinetics and affinity parameters and standard deviations are shown in the table where n is the number of replicates, k_a is the association rate constant, k_d is the dissociation rate constant, and K_D is the equilibrium dissociation constant. N.D. is not determined.

scFv	n	k_a (1/Ms)	k_d (1/s)	K_D (nM)
M2J1-scFv (CAA)	6	9.13E+04 ± 5.16E+03	1.38E-02 ± 5.29E-04	152 ± 10.4
M2J1-CAS-scFv	6	N.D.	N.D.	1440 ± 113
M2J1-CAN-scFv	5	N.D.	N.D.	19300 ± 1760
M2J1-CAT-scFv	6	N.D.	N.D.	> 25000
M2J1-CAR-scFv	5	N.D.	N.D.	> 25000

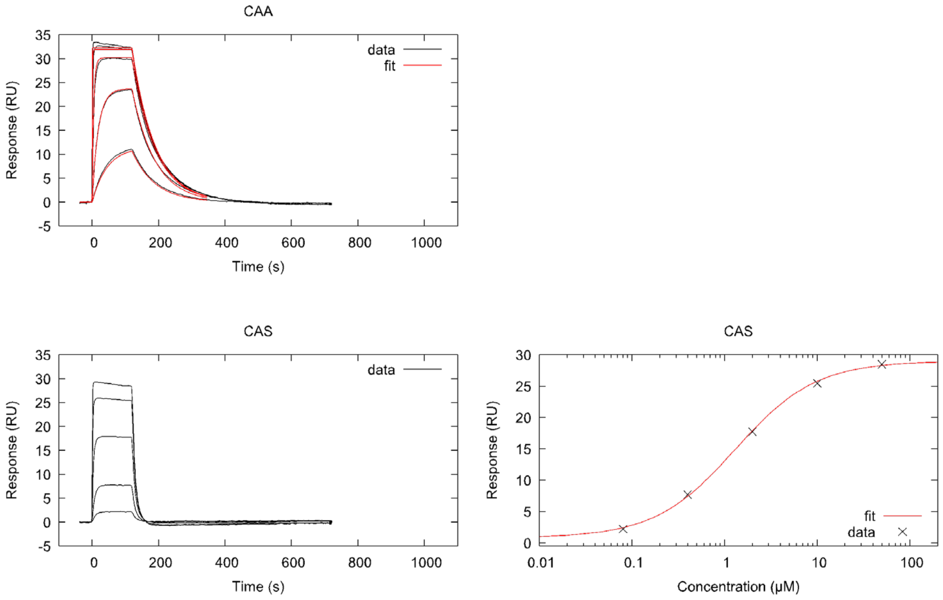

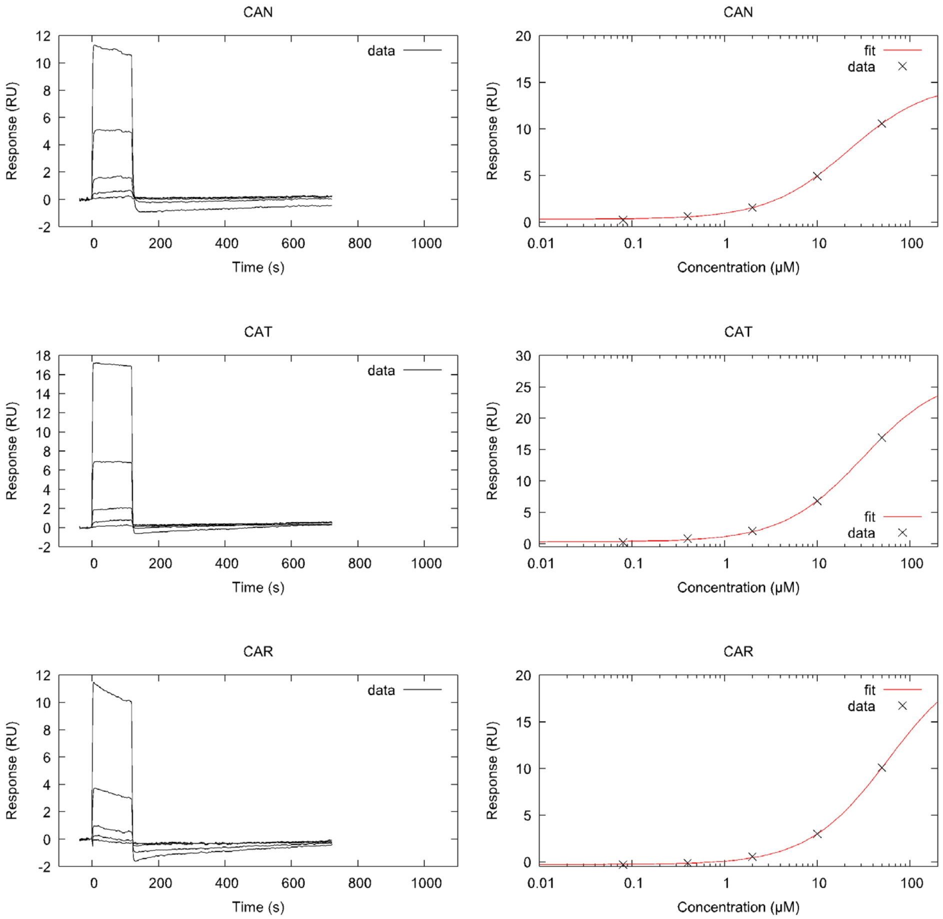
CAN
Response (RU)
Time (s)
data
CAN
Response (RU)
Concentration (μM)
fit
data
CAT
Response (RU)
Time (s)
data
CAT
Response (RU)
Concentration (μM)
fit
data
CAR
Response (RU)
Time (s)
data
CAR
Response (RU)
Concentration (μM)
fit
data

Supplementary Figure 3. Binding kinetics of anti-CD33 antibodies.

a, SPR sensorgrams for a conventional anti-CD33 scFv-Fc (M195), conditional scFv-Fcs with intermediate (P01_A09) and high MTX sensitivity (P02_D09). Sensorgrams show binding of 200 nM hCD33 analyte binding to captured scFv-Fc in the absence (black curve) or presence of 10 µM MTX (red curve). b, Table showing SPR binding responses for hCD33 in the absence ($Response_{hCD33}$) or presence of 10 µM MTX ($Response_{hCD33+10\mu M\ MTX}$). Responses were taken at 5 seconds before the end of the analyte injection (55 second time point). Percent inhibition was calculated as 100 * ($Response_{hCD33}$ - $Response_{hCD33+10\mu M\ MTX}$) / $Response_{hCD33}$. c, Sensorgrams showing the effect of varying the MTX concentration in the hCD33 analyte samples (MTX concentrations are given in the legend, the hCD33 concentration was 200 nM). All MTX inhibition experiments (panels a, b and c) were done using an A-B-A injection type on a Biacore 8K SPR biosensor. For analyte cycles containing MTX, the pre-analyte/post-analyte solution A contained MTX at the specified concentration allowing MTX binding to come to equilibrium before association of the hCD33 + MTX containing solution B. d, Kinetic and affinity parameters at 37°C for direct binding of hCD33 to captured scFv-Fcs (without MTX), as determined by SPR. e, Sensorgrams and fits for hCD33 binding to captured conventional scFv-Fc, and conditional scFv-Fcs (P01_A09, P02_D09). The hCD33 antigen was captured at low density onto a C1 sensor surface, and scFv-Fcs were flowed as analytes at binding sites concentrations of 1.6, 8, 40, 200 and 1000 nM. f, Kinetics and affinity parameters at 37°C for direct binding of MTX to immobilized scFv-Fcs, as determined by SPR. N.D. is not determined. g, Sensorgrams and fits for MTX binding to captured conventional scFv-Fc, and conditional scFv-Fcs (P01_A09, P02_D09). Binding of P01_A09 exhibited fast binding kinetics, and the affinity was determined by the steady-state binding responses (steady state fit is shown in the inset). For experiments with n > 1, standard deviations are shown. n is the number of replicates, k_a is the association rate constant, k_d is the dissociation rate constant, and K_D is the equilibrium dissociation constant.

a

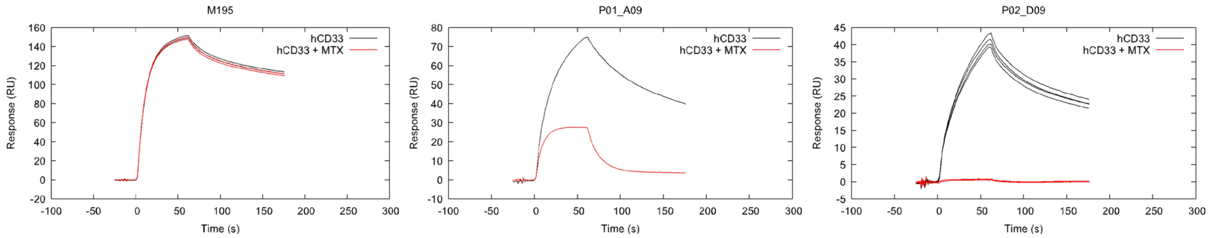

b

scFv	n	Response$_{hCD33}$ (RU)	Response$_{hCD33+10\mu M\ MTX}$ (RU)	% inhibition (10 μM MTX)
M195	2	150 ± 2.03	148 ± 1.74	0.978
P01_A09	1	74.3	27.5	63.0
P01_F05	1	74.2	0.186	99.7
P02_A10	1	36.9	1.30	96.5
P02_B03	1	23.2	2.89	87.6
P02_D08	1	83.6	37.2	55.5
P02_D09	4	40.5 ± 1.75	0.654 ± 0.102	98.4
P02_E08	1	23.3	2.02	91.3
P02_E11	1	87.4	4.37	95.0
P03_A01	1	53.9	5.50	89.8
P03_G12	1	63.2	0.593	99.1
P03_H04	1	59.7	0.321	99.5
P04_C09	1	53.4	15.1	71.8
P07_C04	1	106	81.2	23.3
P08_C08	1	81.3	39.5	51.4

c

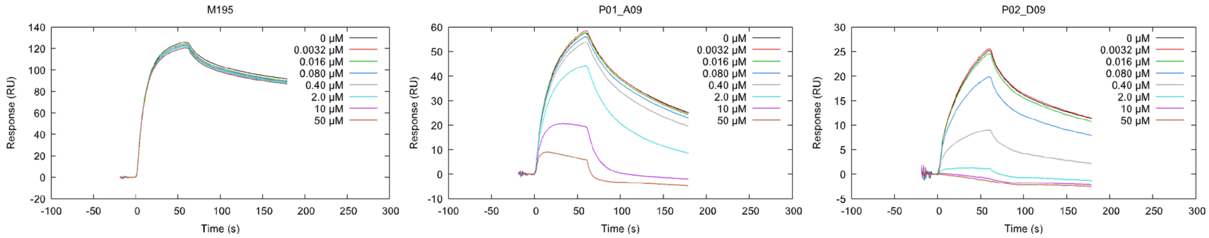

d

scFv	CD33 binding			
	n	k_a (1/Ms)	k_d (1/s)	K_D (nM)
M195	3	1.23E+06 ± 2.73E+04	3.26E-04 ± 6.86E-06	0.265 ± 0.00812
P01_A09	5	3.43E+06 ± 3.96E+05	5.22E-03 ± 3.02E-04	1.53 ± 0.198
P01_F05	5	1.22E+06 ± 1.03E+05	3.80E-03 ± 2.51E-04	3.15 ± 0.338
P02_A10	5	3.33E+06 ± 1.06E+06	1.86E-02 ± 4.78E-03	5.67 ± 2.32
P02_B03	3	5.21E+05 ± 2.03E+04	1.42E-02 ± 9.07E-04	27.4 ± 2.05
P02_D08	5	2.68E+06 ± 1.34E+05	2.92E-03 ± 1.97E-04	1.09 ± 0.0914
P02_D09	4	3.64E+05 ± 2.82E+04	9.71E-03 ± 2.31E-03	26.7 ± 6.67
P02_E08	5	6.27E+04 ± 1.54E+04	4.94E-03 ± 1.85E-03	84.4 ± 37.8
P02_E11	5	1.62E+06 ± 6.72E+04	1.71E-03 ± 6.04E-05	1.06 ± 0.0574
P03_A01	5	1.80E+06 ± 1.46E+05	2.55E-03 ± 2.36E-04	1.42 ± 0.175
P03_G12	5	1.68E+06 ± 1.64E+05	3.78E-03 ± 3.14E-04	2.25 ± 0.288
P03_H04	3	9.18E+05 ± 6.03E+04	2.09E-02 ± 2.60E-03	22.7 ± 3.20
P04_C09	5	6.15E+05 ± 3.72E+04	3.69E-04 ± 8.31E-05	0.598 ± 0.139
P07_C04	5	3.55E+06 ± 2.42E+05	1.18E-03 ± 2.20E-05	0.334 ± 0.0237
P08_C08	5	2.27E+06 ± 9.21E+04	2.23E-03 ± 1.28E-04	0.983 ± 0.0693

e

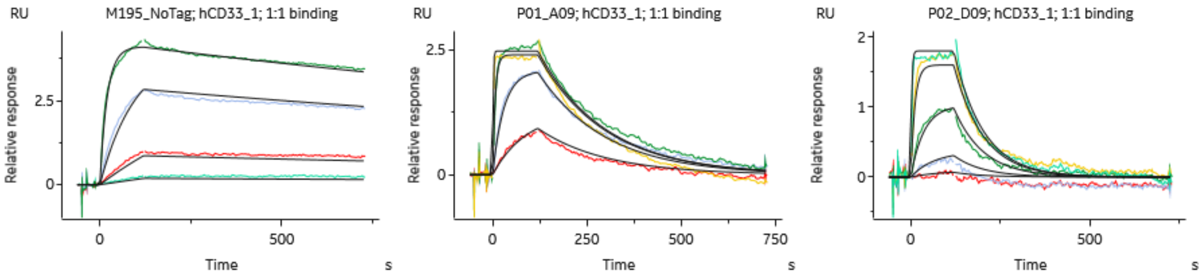

f

scFv	MTX binding			
	n	k_a (1/Ms)	k_d (1/s)	K_D (nM)
M195	30	*no binding*	*no binding*	*no binding*
P01_A09	11	N.D.	N.D.	4500 ± 496
P01_F05	9	5.54E+04 ± 2.07E+03	1.37E-02 ± 6.81E-04	248 ± 15.4
P02_A10	10	1.44E+04 ± 1.06E+03	2.44E-02 ± 3.18E-03	1700 ± 254
P02_B03	11	1.55E+04 ± 7.16E+03	7.66E-02 ± 2.93E-02	5060 ± 3030
P02_D08	9	4.92E+04 ± 3.99E+03	3.27E-02 ± 3.40E-03	665 ± 87.6
P02_D09	24	3.39E+04 ± 2.15E+03	2.12E-02 ± 7.37E-04	626 ± 45.2
P02_E08	9	N.D.	N.D.	2370 ± 166
P02_E11	9	8.09E+04 ± 9.62E+03	2.28E-02 ± 3.09E-03	283 ± 51.1
P03_A01	6	N.D.	N.D.	1280 ± 217
P03_G12	11	9.26E+04 ± 5.46E+03	1.48E-02 ± 1.58E-03	160 ± 19.5
P03_H04	8	4.26E+04 ± 1.87E+03	3.94E-02 ± 1.47E-03	926 ± 53.3
P04_C09	9	4.67E+04 ± 4.97E+03	2.13E-02 ± 2.47E-03	456 ± 71.9
P07_C04	8	5.60E+04 ± 7.11E+03	4.53E-03 ± 4.83E-04	81.9 ± 13.6
P08_C08	11	N.D.	N.D.	2400 ± 139

g

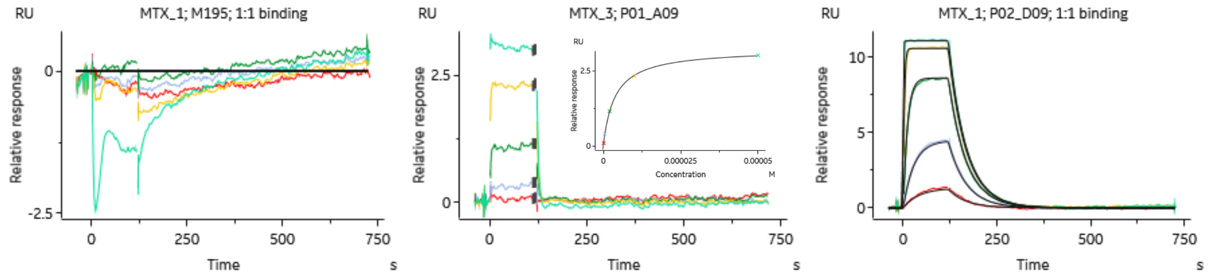

Supplementary Figure 4. Binding of anti-EGFR antibodies.

a, Surface plasmon resonance sensorgrams for an anti-EGFR scFv-Fc with low MTX sensitivity (B10), intermediate MTX sensitivity (A06) and high MTX sensitivity (A01). Sensorgrams show binding of 300 nM hEGFR analyte binding to captured scFv-Fc in the absence (hEGFR, black curve) or presence of 10 μM MTX (hEGFR + MTX, red curve). b, Table showing binding responses (as determined by SPR) for hEGFR in the absence ($\text{Response}_{\text{hEGFR}}$) or presence of 10 μM MTX ($\text{Response}_{\text{hEGFR+10μM MTX}}$). Responses were taken at 5 seconds before the end of the analyte injection (115 s time point as shown in panel A). Percent inhibition (% inhibition) was calculated as 100 * ($\text{Response}_{\text{hEGFR}}$ - $\text{Response}_{\text{hEGFR+10μM MTX}}$) / $\text{Response}_{\text{hEGFR}}$.

a

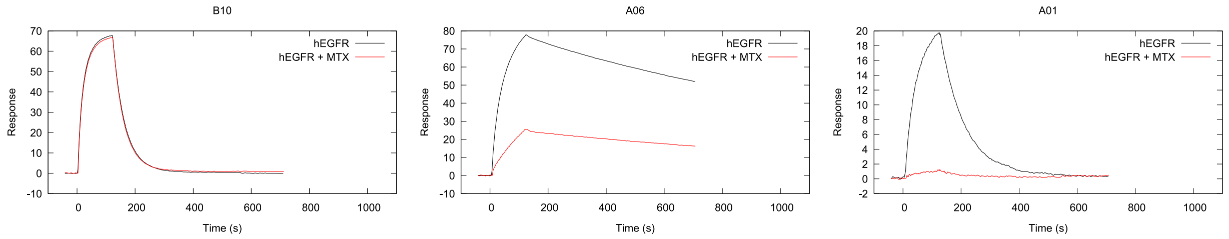

b

scFv	n	Response$_{hEGFR}$ (RU)	Response$_{hEGFR+10\ \mu M\ MTX}$ (RU)	% inhibition (10 μM MTX)
A01	1	19.4	0.979	95.0
A04	1	7.78	0.392	95.0
A05	1	41.2	4.31	89.6
A06	1	76.2	24.53	67.8
A10	1	10.9	4.59	57.9
A12	1	32.6	29.1	10.7
B05	1	31.9	16.1	49.5
B07	1	7.88	8.26	-4.80
B10	1	67.5	66.6	1.41
B12	1	27.5	25.0	8.96
C01	1	6.10	6.44	-5.50
C02	1	5.78	6.32	-9.33
C08	1	8.33	7.48	10.2
C09	1	33.5	29.7	11.2
C10	1	5.19	3.85	25.8
C12	1	36.0	5.88	83.7
D01	1	27.3	16.7	38.9
D02	1	20.5	7.90	61.4
D05	1	17.1	10.36	39.4
D06	1	5.82	3.13	46.2
D08	1	5.63	3.00	46.7
D09	1	21.6	4.69	78.3
D12	1	10.0	3.36	66.4

Supplementary Figure 5. Contributions of CDRH2 to MTX binding.

Backbone carbonyls from Ser52 and Tyr53 of CDRH2 (yellow) form hydrogen bonds with Arg72 and Asn74 (teal) of FW3, helping orient the side chains for hydrogen bonding with the pterin moiety of MTX (pink). CDRH1 has been removed from the view for clarity.

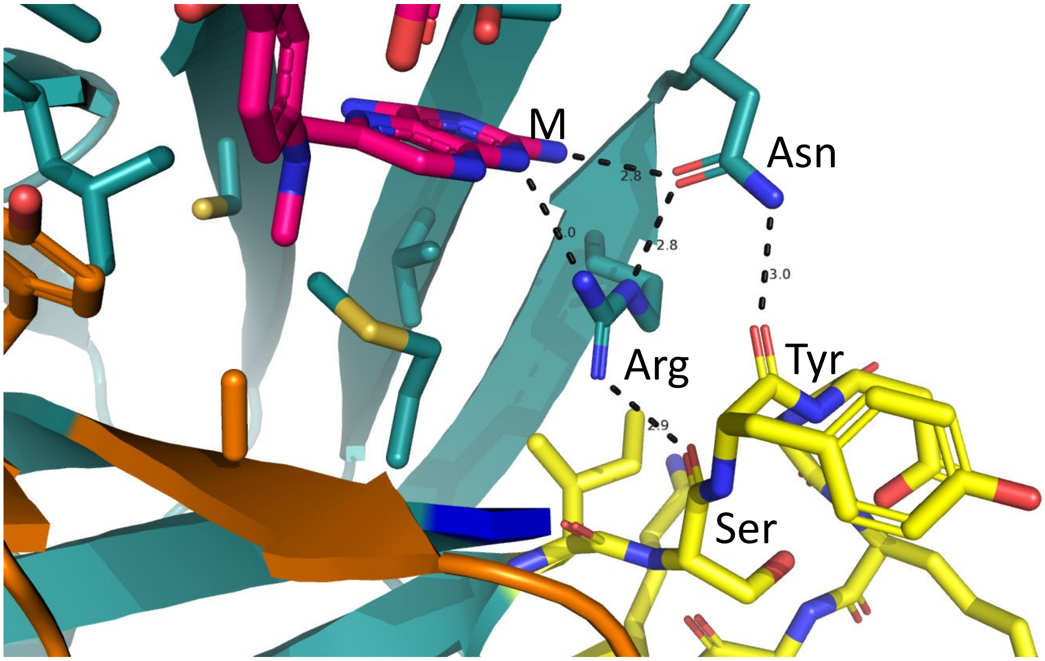

Supplementary Figure 6. CAR T cells based on conditional scFvs can be readily made. Primary T cell transduction levels (% shown as numbers in dot plots) for CARs based on a panel of conditional scFvs that target CD33 were determined by flow cytometry staining for the V5 tag. A CAR designed using the conventional anti-human CD33 antibody M195 (CAR) was used as a comparator.

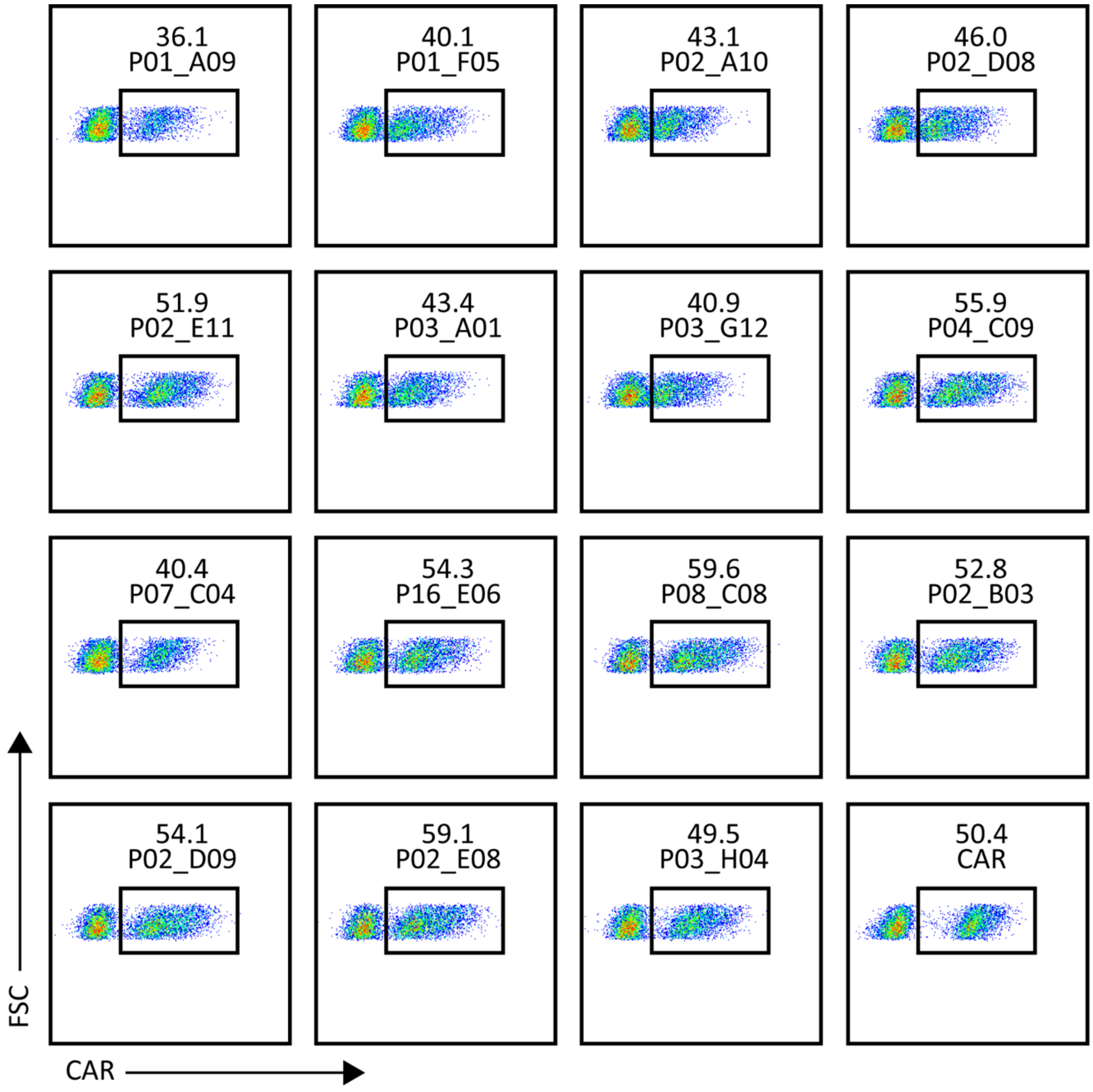

Supplementary Figure 7. CondCAR T cells exhibit target-specific cytotoxicity.

Flow cytometry measurements of CD33 expression on the surface of K562 (top left) and MV4-11 (top right) show CD33 expression on MV4-11 (orange: anti-human CD33 antibody, blue: isotype control, red: no antibody label). CondCAR T cells perform selective lysis of MV4-11 cells at a comparable level as CD33-specific conventional CAR T cells. Results are normalized to NTD (n=3 biologically independent samples, mean ± SD). E:T = 1:1.

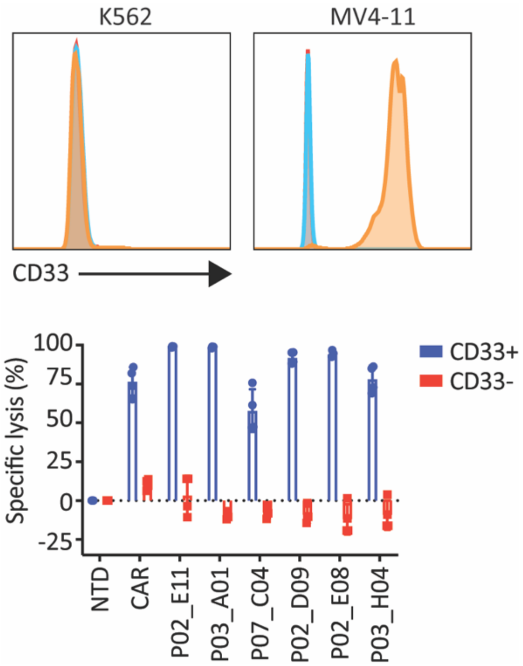

Supplementary Figure 8. Leucovorin supplementation and overexpression of MTX-resistant DHFR variant (DHFRmut) can mitigate MTX-associated toxicity.

The reduction of MTX-associated toxicity using leucovorin (ten-times molar quantity of MTX) and/or overexpression of DHFRmut on T cells and MV4-11 cells was assessed over a range of MTX concentrations relevant to inhibition of condCAR T cell activity. CAR T cells (in culture with anti-CD3 and anti-CD28 coated beads) and MV4-11 cells (in culture) were exposed to MTX for 48 hours prior to quantifying viability (normalized to cell viability at 0 μM MTX; n=2 independent experiments). CAR T viability was quantified via flow cytometry-based cell enumeration using CountBright^{TM} beads. MV4-11 cell viability was quantified by firefly luciferase activity (n=2 independent experiments).

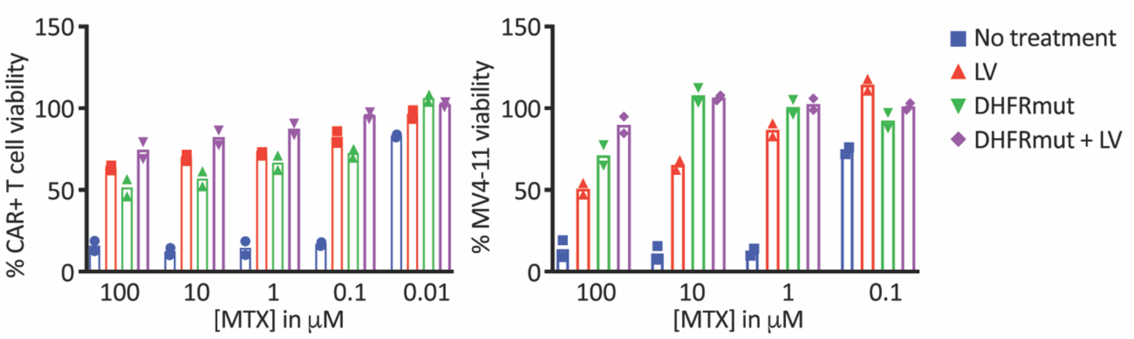

Supplementary Figure 9. CondCAR T activity changes rapidly over a narrow concentration range of MTX.

Target lysis by condCAR T cells (P02_D09-CAR) and conventional CAR T cells were measured following a 48-hour co-culture at MTX concentrations ranging from 0.39 to 100 μM. E:T = 1:1 (mean ± SD; n=3 independent experiments).

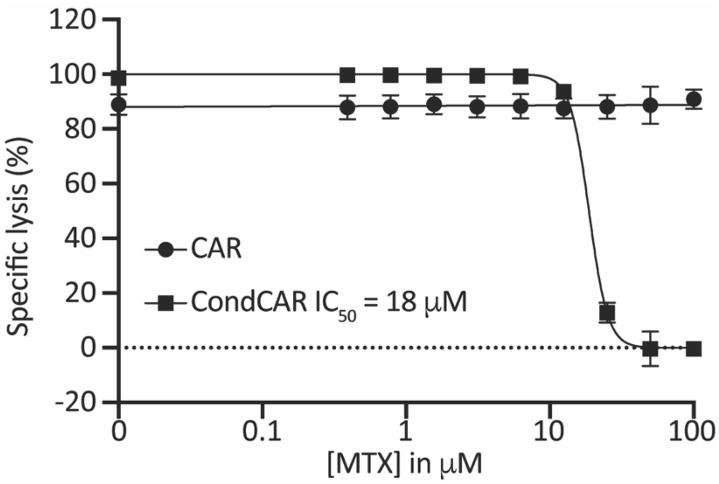

Supplementary Figure 10. Target-specific lysis of EGFR-expressing tumor cells by condCAR T cells is dependent on the presence of MTX.

a, Primary T cell transduction levels (% shown as numbers in dot plots) for CARs based on a panel of conditional scFvs that target EGFR were determined by flow cytometry detection of CAR using recombinant human EGFR conjugated to PE. A CAR designed using the conventional anti-human EGFR antibody, Erbitux, was used as a comparator. b, CondCAR T cells perform selective lysis of U87-EGFR (EGFR+) cells at a comparable level as EGFR-specific conventional CAR T cells. Results are normalized to NTD. c. Target cell lysis was determined after a 48hr co-incubation of U87-EGFR target cells with conventional CAR T cells or condCAR T cells. Effector:target (E:T) = 1:1 (n=3 biologically independent samples, mean ± SD). P-values were calculated by paired two-tailed t-test; N.S., non-significant (p>0.01), **p<0.01; ***p<0.001.)

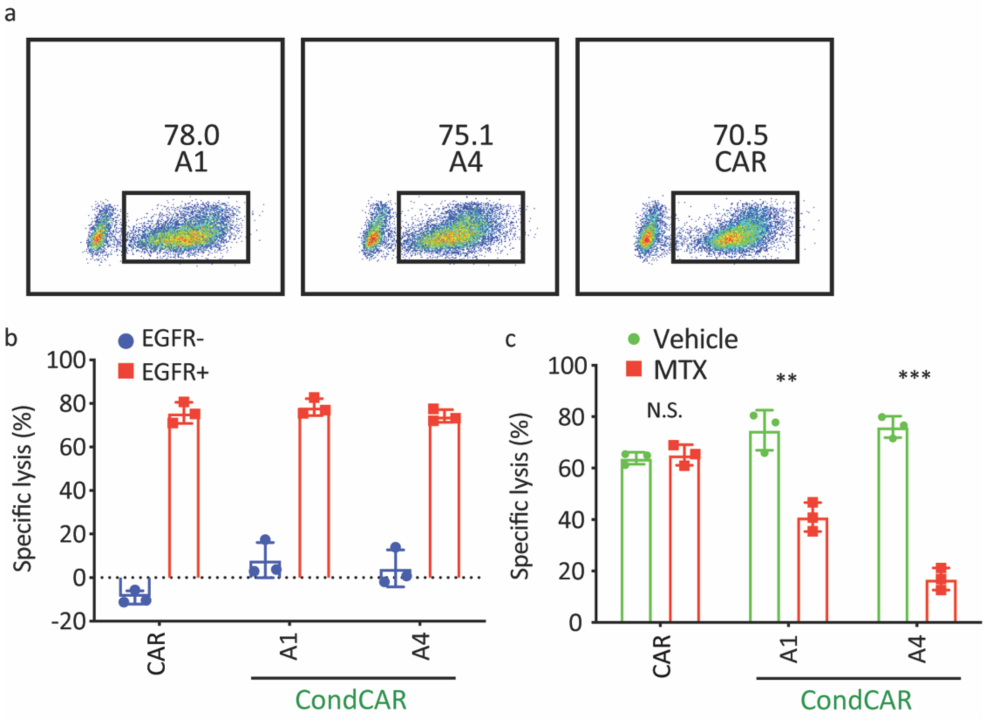

Supplementary Figure 11. Tumor growth inhibition by condCAR T cells is delayed in the presence of MTX.

Bioluminescence analysis of mice infused by condCAR T cells (P02_D09-CAR T cells), conventional CAR T cells, or NTD T cells with 0 or 250 mg/mouse/day MTX. Mice with established MV4-11mut tumor burden received equal number of conventional CAR T cells, condCAR T cells, or non-transduced T cells (NTD) normalized to the lowest %CAR+ value. All images illustrate the radiance (p/s/cm^2/sr) of luminescence.

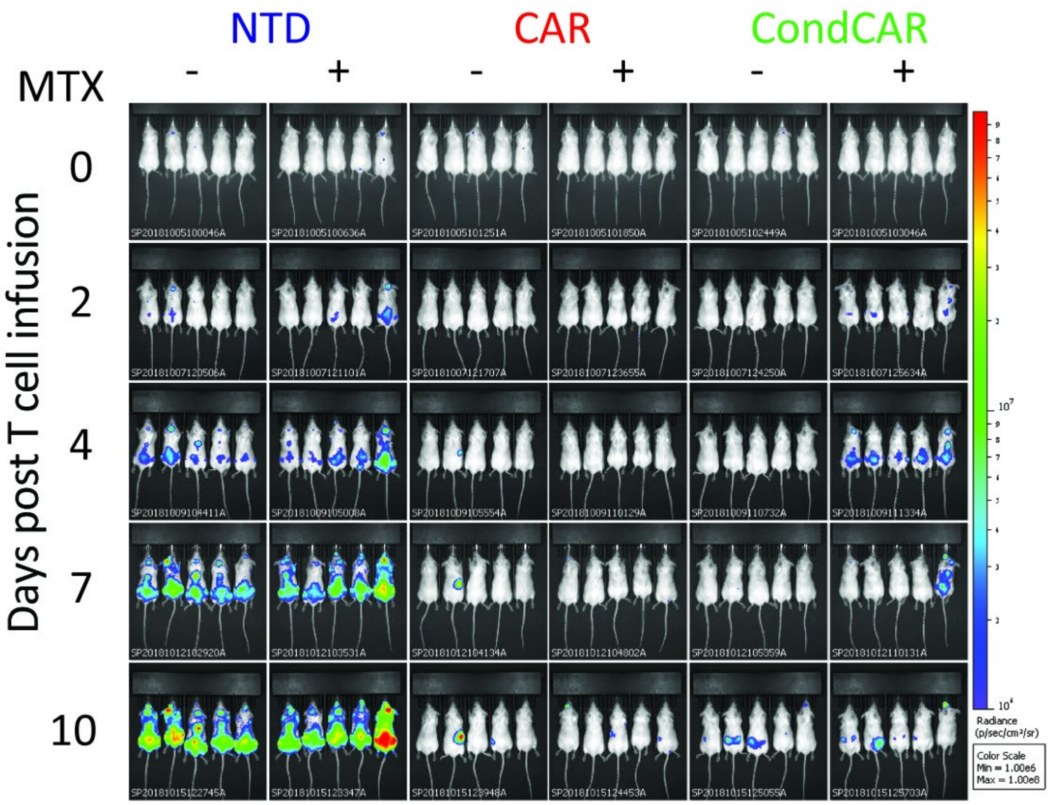

Supplementary Figure 12. MTX analogues with reduced toxicity.

MTX analogues (PF-07091181 in red and PF-07067428 in blue) can prevent toxicity to condCAR T cells (P02_D09-CAR T cells) while maintaining inhibition of target cell lysis. CondCAR T viability over a range of MTX analogue concentrations was quantified via flow cytometry-based cell enumeration using CountBright beads. The inhibitory effect of MTX analogues on condCAR T cell's antigen-specific lysis was assessed over the same range of concentrations. E:T = 1:1 (n=1).

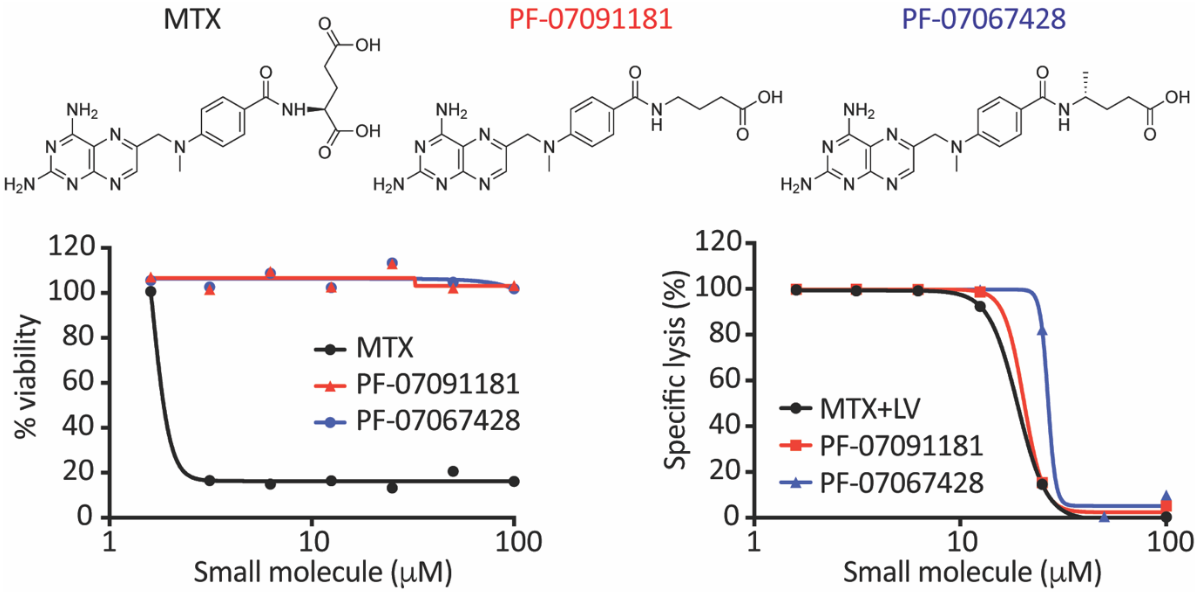

Supplementary Figure 13. Conditional scFv and CAR T cell screening.

a, Schematic of the conditional scFv discovery process for selecting highly specific clones. In the CD33 campaign, phage displaying clonal scFvs were screened by ELISA for direct binding to immobilized biotinylated targets (human CD33 (hCD33), mouse 4-1BB (m4-1BB), and MTX) and blank streptavidin wells. CD33-specific clones were then screened in inhibition ELISAs, where phage binding to CD33 was tested in buffer or mixed with soluble inhibitor (100 nM hCD33, or 10 µM MTX). Percent inhibition by CD33 was used to affinity rank and further establish CD33 binding specificity. MTX-sensitive scFvs were cloned into a CAR lentiviral vector. b, Direct binding ELISA results; binding response to m4-1BB negative control antigen is plotted versus binding to hCD33 (top), MTX (middle) or blank (bottom) c, Inhibition ELISA results; phage binding to immobilized hCD33 with and without soluble inhibitor - MTX (left) and hCD33 (right); blue and red lines show 0% and 50% inhibition, respectively. b, c, black points show clones selected for further characterization (listed in Supplementary Figure 3b, d, f), other clones are shown in grey. d, Schematic outline of production process for selecting highly specific, well-behaved CAR T cell clones. Healthy donor T cells were stimulated for two days prior to transduction with the lentiviral vectors described above and one encoding a conventional CAR. Nine days following transduction, surface activation markers (CD25 & CD137) were measured to determine tonic signaling levels. On day 14, prior to cryopreservation of the prepared T cells, CAR transduction efficiencies and the degree of T cell differentiation (CD62L & CD45RO) were quantified. Cell expansion was quantified throughout production. e, CondCAR T cell clones exhibit similar expansion capacity as conventional CAR T cells. f, CAR transduction efficiencies were similar across the different condCARs and the conventional CAR. g, CondCARs confer low levels of tonic signaling in T cells that is comparable to the conventional CAR. h, T cell differentiation states of the condCAR T cell clones and the conventional CAR T cells are similar at the end of the CAR T cell production process.

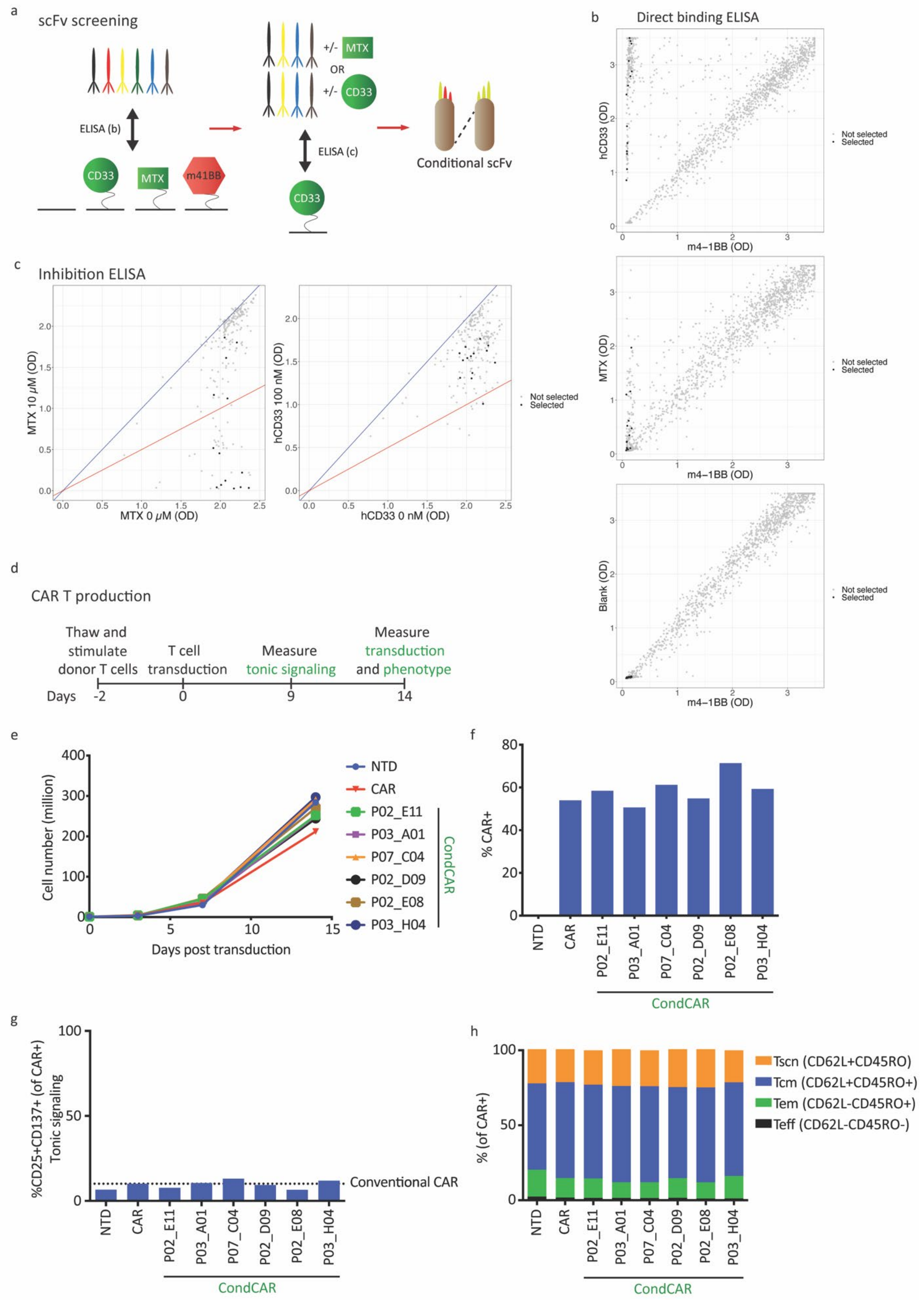
a
scFv screening
ELISA (b)
CD33
MTX
m41BB
+/- MTX
OR
+/- CD33
ELISA (c)
Conditional scFv
b
Direct binding ELISA
hCD33 (OD)
m4−1BB (OD)
MTX (OD)
Blank (OD)
Not selected
Selected
c
Inhibition ELISA
MTX 10 μM (OD)
MTX 0 μM (OD)
hCD33 100 nM (OD)
hCD33 0 nM (OD)
d
CAR T production
Thaw and stimulate donor T cells
T cell transduction
Measure tonic signaling
Measure transduction and phenotype
Days
-2
0
9
14
e
Cell number (million)
Days post transduction
NTD
CAR
P02_E11
P03_A01
P07_C04
P02_D09
P02_E08
P03_H04
CondCAR
f
% CAR+
g
%CD25+CD137+ (of CAR+)
Tonic signaling
Conventional CAR
h
% (of CAR+)
Tscn (CD62L+CD45RO)
Tcm (CD62L+CD45RO+)
Tem (CD62L-CD45RO+)
Teff (CD62L-CD45RO-)

Supplementary Figure 14. Gating strategies used for flow cytometry analysis.

a, gating strategy to measure antigen expression. b, gating strategy to measure CAR expression and induction of CD69 following antigen stimulation. c, gating strategy to measure antigen-dependent proliferation. d, gating strategy to measure CAR expression, tonic signaling, and phenotype of CAR T cells at the end of production. e, gating strategy to analyze CAR T cells from mouse blood.

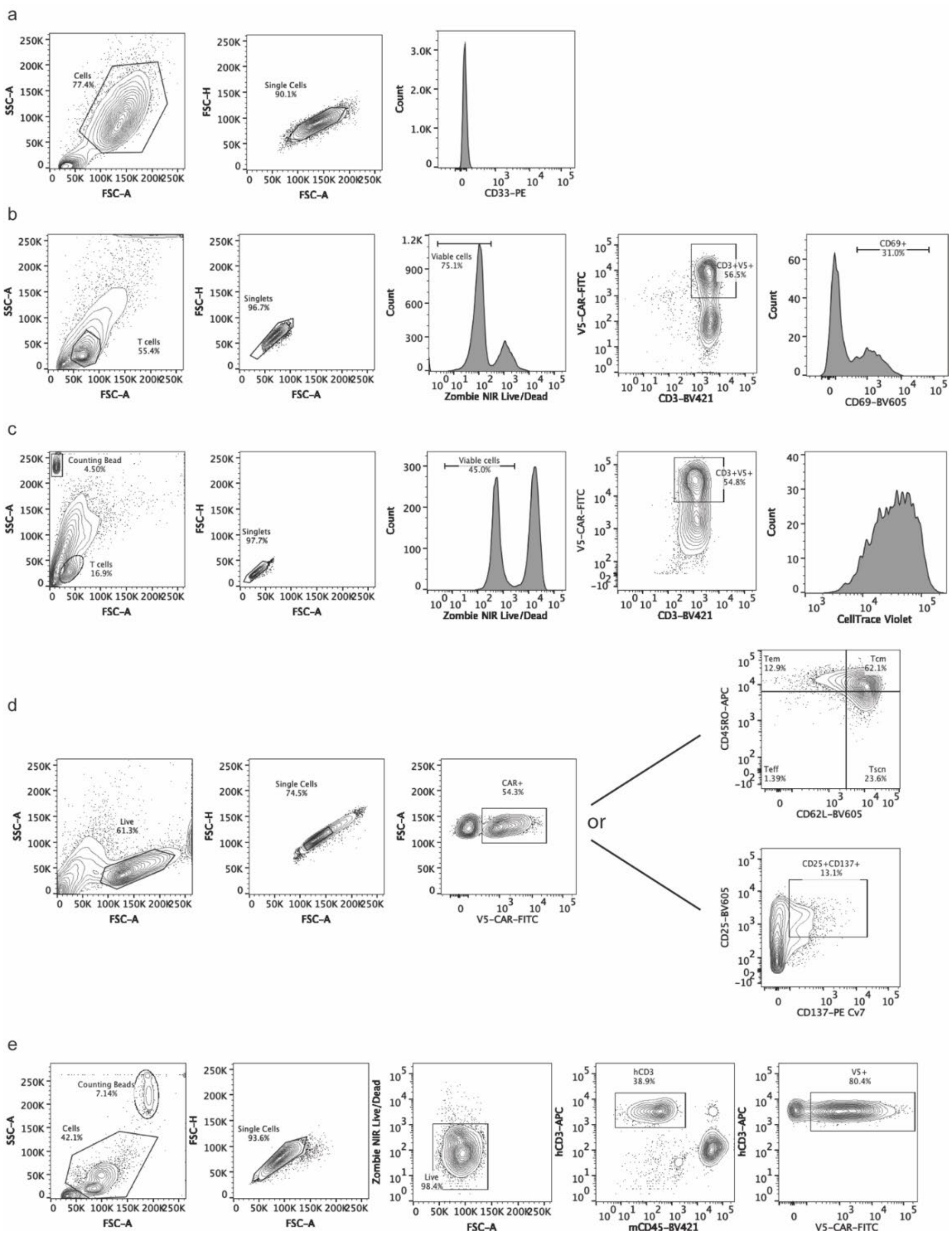

Supplementary Tables

Supplementary Table 1: Kinetics and affinity parameters of MTX analogues binding to immobilized scFv-Fcs at 37°C.

a

scFv	PF-07091181 binding kinetics				
	n	k_a (1/Ms)	k_d (1/s)	K_D (nM)	$K_D/K_{D, MTX}$
M195	10	*no binding*	*no binding*	*no binding*	N.D.
P01_A09	3	N.D.	N.D.	12300 ± 2990	2.73
P01_F05	3	3.45E+04 ± 3.10E+03	1.61E-02 ± 2.29E-03	472 ± 79.5	1.90
P02_A10	4	1.01E+04 ± 1.51E+03	2.11E-02 ± 3.33E-03	2090 ± 455	1.23
P02_B03	4	9.84E+03 ± 4.04E+03	7.24E-02 ± 2.51E-02	7510 ± 4030	1.48
P02_D08	4	2.15E+04 ± 3.41E+03	2.91E-02 ± 7.20E-04	1380 ± 222	2.08
P02_D09	10	1.92E+04 ± 1.65E+03	1.84E-02 ± 6.07E-04	961 ± 88.3	1.54
P02_E08	3	N.D.	N.D.	3650 ± 640	1.54
P02_E11	3	4.15E+04 ± 2.39E+03	1.97E-02 ± 8.39E-04	477 ± 34.1	1.69
P03_A01	3	N.D.	N.D.	2270 ± 768	1.77
P03_G12	4	5.67E+04 ± 5.31E+03	1.64E-02 ± 6.22E-04	292 ± 29.5	1.83
P03_H04	3	2.28E+04 ± 2.95E+03	4.68E-02 ± 1.10E-03	2070 ± 273	2.24
P04_C09	3	2.75E+04 ± 5.86E+03	2.84E-02 ± 2.15E-03	1070 ± 243	2.35
P07_C04	4	2.97E+04 ± 4.16E+03	4.55E-03 ± 1.35E-04	156 ± 22.3	1.90
P08_C08	3	N.D.	N.D.	6300 ± 1580	2.63

b

scFv	PF-07067428 binding kinetics				
	n	k_a (1/Ms)	k_d (1/s)	K_D (nM)	$K_D/K_{D,\ MTX}$
M195	10	*no binding*	*no binding*	*no binding*	N.D.
P01_A09	3	N.D.	N.D.	19700 ± 5320	4.38
P01_F05	3	3.43E+04 ± 2.90E+03	2.63E-02 ± 1.13E-03	772 ± 73.2	3.11
P02_A10	4	1.30E+04 ± 1.79E+03	4.10E-02 ± 4.34E-03	3180 ± 551	1.87
P02_B03	4	1.17E+04 ± 5.61E+03	1.32E-01 ± 3.85E-02	12300 ± 6870	2.43
P02_D08	4	2.93E+04 ± 2.16E+03	4.98E-02 ± 1.05E-03	1700 ± 130	2.56
P02_D09	10	2.14E+04 ± 1.01E+03	3.33E-02 ± 1.25E-03	1560 ± 94.5	2.49
P02_E08	3	N.D.	N.D.	6130 ± 123	2.59
P02_E11	3	5.14E+04 ± 3.01E+03	2.96E-02 ± 1.07E-03	578 ± 39.7	2.04
P03_A01	3	N.D.	N.D.	4170 ± 776	3.26
P03_G12	4	5.85E+04 ± 6.74E+03	2.38E-02 ± 7.30E-04	411 ± 49.0	2.57
P03_H04	3	2.71E+04 ± 8.69E+02	5.91E-02 ± 9.51E-04	2180 ± 78.4	2.35
P04_C09	5	3.87E+04 ± 9.00E+03	4.91E-02 ± 6.55E-03	1300 ± 349	2.85
P07_C04	4	3.99E+04 ± 3.67E+03	7.23E-03 ± 8.36E-04	181 ± 26.7	2.21
P08_C08	3	N.D.	N.D.	8390 ± 1800	3.50

a, Kinetics and affinity parameters at 37°C for direct binding of PF-07091181 to immobilized scFv-Fcs, as determined by surface plasmon resonance. b, Kinetics and affinity parameters at 37°C for direct binding of PF-07067428 to immobilized scFv-Fcs, as determined by surface plasmon resonance. n is the number of replicates, k_a is the association rate constant, k_d is the dissociation rate constant, and K_D is the equilibrium dissociation constant. Standard deviations are shown for each parameter. $K_D/K_{D,\ MTX}$ is the ratio of the equilibrium dissociation constant for the MTX-analogue to that of MTX (mean K_D values were used). N.D. is not determined. See the supplemental methods for more details related to surface plasmon resonance experiments.

Supplementary Table 2: Crystallographic data collection and refinement statistics.

	MTX bound Conditional scFv	Apo Conditional scFv
Data Collection		
Wavelength (Å)	1	0.9774
Space group	$I2_13$	$P2_1$
Cell dimensions a/b/c (Å)	118.99, 118.99, 118.99	81.23 104.64 87.92
α, β, γ(°)	90, 90, 90	90, 113.57, 90
Resolution (Å)	59.5 – 2.0 (2.073-2.0)	43.88 – 2.20 (2.8-2.2)
R_{pim} (%)	2.86 (53.94)	7.86 (52.98)
Mn (I/sI)	21.68 (2.42)	6.9 (7.0)
$CC_{1/2}$	0.999 (0.645)	0.999 (0.881)
Completeness (%)	99.26 (100)	99.92 (99.88)
Redundancy	19.0 (20.3)	6.2 (537)
Unique reflections	18943 (1883)	68385 (6782)
Refinement		
R_{work} / R_{free} (%)	18.88 (25.53) / 22.07 (31.43)	19.84 (27.81) / 23.70 (29.74)
Protein chains in AU	1	4
No. of protein atoms	1769	6772
No. of ligand atoms	62	166
No. of solvent molecules	149	707
Average B-factor	40.43	33.58
Protein	39.19	32.63
Ligands	27.31	49.48
Solvent	48.1	38.87
RMSD bond lengths (Å)	0.002	0.003
RMSD angles (°)	0.52	0.61
Ramachandran best / disallowed (%)	98.28 / 0	97.86 / 0

*Values in parentheses are for highest-resolution shell.

Supplementary Methods

Analysis of scFvs/human CD33 interactions at 37°C.

This method describes the analysis of various scFvs binding to human CD33 at 37°C. Experiments were performed on a Biacore 8K Surface Plasmon Resonance based biosensor (GE Healthcare Lifesciences, Marlborough, MA).

Anti-His tag sensor chips were prepared at 25°C with a running buffer of 10 mM HEPES, 150 mM NaCl, 0.05% (v/v) Tween-20, pH 7.4. All surfaces of a Biacore C1 sensor chip were pre-conditioned with four 60-second injections of a 0.1 M Glycine pH 12,0.3% Triton 100 mixture, then activated with a 1:1 (v/v) mixture of 400 mM EDC and 100 mM NHS for 7 minutes at flow rate of 10 µL/min. An anti-His reagent (His-tag antibody, R&D Systems Catalog #MAB050) was diluted to 50 µg/mL in 10 mM sodium acetate (pH 5.0) and injected on both flow cells of all eight channels for 7 minutes at 10 µL/min. All flow cells were blocked with 100 mM ethylenediamine in 200 mM Borate buffer pH 8.5 for 7 minutes at 10 µL/min.

All interaction experiments were performed at 37°C using a running buffer of 10 mM HEPES, 150 mM NaCl, 1 mg/mL BSA, 0.05% Tween20, pH 7.4. In order to remove avidity effects, a minimal amount of CD33 was captured on the anti-HIS C1 chip surface. These conditions gave homogeneous binding kinetics while the opposite orientation, where scFv was capture by anti-human Fc and CD33 flowed as the analyte, gave heterogeneous kinetics (data not shown). The His-tagged human CD33 antigen was captured at low concentration (0.6 µg/mL to 1.2 µg/mL depending on the activity of the anti-HIS surface) onto flow cell 2 of all eight channels at a flow rate of 10 µL/min for 2 minutes. Following capture of human CD33 antigen, analyte (buffer, 1.6 nM, 8 nM, 40 nM, 200 nM and/or 1000 nM scFvs; concentrations are in binding sites) was injected over both flow cells of different channels for 2 minutes at a flow rate of 30 µL/min. After each

analyte injection, dissociation was monitored for 10 minutes followed by regeneration of all flow cells with two 30-second injections of 10 mM Glycine at pH 1.7. Buffer cycles were collected for each scFv for double-referencing purposes (double-referencing as previously described[2]). For kinetic analysis, the double-referenced sensorgrams were fit globally to a simple 1:1 Langmuir with mass transport binding model using Biacore 8K Evaluation Software version 1.1.1.7442. For steady-state affinity analysis, the double-referenced equilibrium binding responses were fit with a 1:1 Langmuir steady-state model using Biacore 8K Evaluation Software version 1.1.1.7442.

Determination of kinetics and affinity of MTX and its analogues/scFv interactions at 37°C.
This method describes the determination of the kinetics and affinity of MTX and its analogues binding to scFvs at 37°C. Experiments were performed on a Biacore 8K or Biacore 4000 Surface Plasmon Resonance based biosensor (GE Healthcare Life Sciences, Marlborough, MA).

All scFvs were biotinylated using 1:1 biotin:protein ratio. Immobilization was performed at 25°C with a running buffer of 10 mM HEPES, 150 mM NaCl, 0.05% (v/v) Tween-20, pH 7.4. All surfaces of a Biacore Streptavidin sensor chip were pre-conditioned with 1M NaCl and 0.05 M NaOH for three consecutive 1-minute injections at a flow rate of 10 µL/min. For immobilization performed on the Biacore 4000, biotinylated scFvs were immobilized on spots 1, 2, 4 and 5 on all four flow cells for 6 minutes. For immobilization performed on Biacore 8K, biotinylated scFvs were immobilized on flow cell 2 of all eight channels for 6 minutes at 10 µL/min. After capture of biotinylated scFvs, all spots (Biacore 4000) or flow cells (Biacore 8K) were blocked with 20µM Amine-PEG2-Biotin (ThermoFisher, Waltham, MA).

All interaction experiments were performed at 37°C using a running buffer of 50 mM Tris, 150 mM NaCl, 0.05% Tween20, 5% DMSO, pH 7.4. Eight solvent correction solutions with DMSO ranging

from 4.5 % to 5.8 % were prepared in running buffer. All eight solvent correction solutions were injected sequentially at 30 µL/min in the first cycle and either every 50 cycles for 30 seconds in Biacore 4000 experiments or every 24 cycles for 20 seconds in Biacore 8K experiments. Analyte (buffer, 0.08 µM, 0.4 µM, 2 µM, 10 µM and 50 µM of MTX or MTX analogues) was injected at a flow rate of 30 µL/min for two minutes in either all four flow cells for Biacore 4000 experiments or all eight channels for Biacore 8K experiments. After each injection, dissociation was monitored for 10 minutes. Spot 3 of all four flow cells on Biacore 4000 and flow cell 1 of all eight channels on Biacore 8K were used as reference surfaces. Buffer cycles were collected for each immobilized biotinylated scFv for double-referencing purposes (double-referencing as previously described[2]). For kinetic analysis performed on Biacore 4000 and Biacore 8K, the solvent-corrected (solvent correction as previously described[3]) double-referenced sensorgrams were fit globally to a simple *1:1 Langmuir with mass transport* binding model using Biacore 4000 Evaluation Software version 1.1 and Biacore 8K Evaluation Software version 1.1.1.7442, respectively. For steady-state affinity analysis performed on Biacore 4000 and Biacore 8K, the solvent-corrected double-referenced equilibrium binding responses were fit with a 1:1 Langmuir steady-state model using Biacore 4000 Evaluation Software version 1.1 and Biacore 8K Evaluation Software version 1.1.1.7442, respectively.

Analysis of scFv/human CD33 interactions in the absence and presence of 10 µM MTX or in the presence of various MTX concentrations at 37°C.

This method describes the analysis of various scFvs binding to human CD33 in the absence and presence of 10 µM MTX or varied MTX concentrations at 37°C. Experiments were performed on a Biacore 8K Surface Plasmon Resonance based biosensor (GE Healthcare Life Sciences, Marlborough, MA).

Anti-human IgG Fc sensor chips were prepared at 25°C with a running buffer of 10 mM HEPES, 150 mM NaCl, 0.05% (v/v) Tween-20, pH 7.4. All surfaces of a Biacore CM4 sensor chip were activated with a 1:1 (v/v) mixture of 400 mM EDC and 100 mM NHS for 7 minutes, at a flow rate of 10 µL/min. An anti-human IgG Fc reagent (Goat Anti-human IgG Fc, Southern Biotech Catalog #2014-01) was diluted to 50 µg/mL in 10 mM sodium acetate (pH 4.5) and injected on all flow cells for 7 minutes at 20 µL/min. All flow cells were blocked with 100 mM ethylenediamine in 200 mM Borate buffer pH 8.5 for 7 minutes at 10 µL/min.

All interaction experiments were performed at 37°C using a running buffer of 10 mM HEPES, 150 mM NaCl, 0.1 mg/mL BSA, 0.05% Tween20, pH 7.4. Each scFv-Fc was captured onto flow cell 2 of each channel at a flow rate of 10 µL/min for 2 minutes. For analyzing the interactions in the absence and presence of 10 µM MTX, pre-analyte A (buffer or 10 µM MTX) followed by analyte (buffer, 200 nM hCD33, 10 µM MTX and a mixture of 200 nM hCD33 and 10 µM MTX) was then injected over both flow cells of all eight channels at a flow rate of 30 µL/min for 2 minutes and 30 seconds, respectively. For analyzing the interactions in the absence and presence of varied MTX concentrations, pre-analyte A (buffer, 0.0032, 0.016, 0.08, 0.4, 2, 10 or 50 µM MTX) followed by analyte (buffer, 200 nM hCD33, a mixture of 200 nM hCD33 and 0.0032, 0.016, 0.08, 0.4, 2, 10 or 50 µM MTX) was injected over both flow cells of all eight channels at a flow rate of 30 µL/min for 2 minutes and 30 seconds, respectively. After analyte injection, dissociation was monitored in pre-analyte A for 1 minutes followed by regeneration of all flow cells with three 60-second injections of 75 mM phosphoric acid. Buffer cycles were collected for each scFv for double-referencing purposes (double-referencing as previously described[2]).

Analysis of scFv/human EGFR interactions in the absence and presence of 10 µM MTX at 37°C. This method describes the analysis of various scFvs binding to human EGFR in the absence and presence of 10 µM MTX at 37°C. Experiments were performed on a Biacore T200 Surface Plasmon Resonance based biosensor (GE Healthcare Life Sciences, Marlborough, MA).
Anti-human IgG Fc sensor chips were prepared at 25°C with a running buffer of 10 mM HEPES, 150 mM NaCl, 0.05% (v/v) Tween-20, pH 7.4. All surfaces of a Biacore CM4 sensor chip were activated with a 1:1 (v/v) mixture of 400 mM EDC and 100 mM NHS for 7 minutes, at a flow rate of 10 µL/min. An anti-human IgG Fc reagent (Goat Anti-human IgG Fc, Southern Biotech Catalog #2014-01) was diluted to 50 µg/mL in 10 mM sodium acetate (pH 4.5) and injected on all flow cells for 7 minutes at 20 µL/min. All flow cells were blocked with 100 mM ethylenediamine in 200 mM Borate buffer pH 8.5 for 7 minutes at 10 µL/min.

All interaction experiments were performed at 37°C. To compare binding in the absence and presence of 10 µM MTX, two kinetics screening experiments were performed. One kinetics screen used 10 mM HEPES, 150 mM NaCl, 0.1 mg/mL BSA, 0.05% Tween20, pH 7.4 (without MTX) as the running and dilution buffer and the other used 10 mM HEPES, 150 mM NaCl, 0.1 mg/mL BSA, 10 µM MTX, 0.05% Tween20, pH 7.4 (with MTX) as the running and dilution buffer. All other experimental conditions were the same. In a given kinetics screen, each scFv-Fc was captured from 2-fold diluted supernatant at a flow rate of 10 µL/min for 2 minutes, onto flow cell 2, 3 or 4 (flow cell 1 was used as a reference surface). Then analyte (buffer or 300 nM hEGFR) was injected over all flow cells at a flow rate of 30 µL/min for 2 minutes and dissociation was monitored for 10 minutes. Then, all flow cells were regenerated with three 30-second injections of 75 mM phosphoric acid. Buffer cycles were collected for each scFv for double-referencing purposes (double-referencing as previously described[2]).

References

1. Van Blarcom, T. et al. Productive common light chain libraries yield diverse panels of high affinity bispecific antibodies. *MAbs* 10, 256-268 (2018).
2. Myszka, D.G. Improving biosensor analysis. *J Mol Recognit* 12, 279-284 (1999).
3. Frostell-Karlsson, A. et al. Biosensor analysis of the interaction between immobilized human serum albumin and drug compounds for prediction of human serum albumin binding levels. *J Med Chem* 43, 1986-1992 (2000).

Full wwPDB X-ray Structure Validation Report ⓘ

Nov 11, 2019 – 02:11 PM EST

PDB ID	:	6UY3
Title	:	Structure of anti-hCD33 conditional scFv with methotrexate
Deposited on	:	2019-11-11
Resolution	:	2.00 Å(reported)

This is a Full wwPDB X-ray Structure Validation Report.

This report is produced by the wwPDB biocuration pipeline after annotation of the structure.

We welcome your comments at *validation@mail.wwpdb.org*
A user guide is available at
https://www.wwpdb.org/validation/2017/XrayValidationReportHelp
with specific help available everywhere you see the ⓘ symbol.

CONFIDENTIAL VALIDATION REPORT

The following versions of software and data (see references ⓘ) were used in the production of this report:

MolProbity	:	4.02b-467
Mogul	:	1.8.0 (224370), CSD as540be (2019)
Xtriage (Phenix)	:	1.13
EDS	:	2.6.1
buster-report	:	1.1.7 (2018)
Percentile statistics	:	20171227.v01 (using entries in the PDB archive December 27th 2017)
Refmac	:	5.8.0158
CCP4	:	7.0.044 (Gargrove)
Ideal geometry (proteins)	:	Engh & Huber (2001)
Ideal geometry (DNA, RNA)	:	Parkinson et al. (1996)
Validation Pipeline (wwPDB-VP)	:	2.6.1

1 Overall quality at a glance (i)

The following experimental techniques were used to determine the structure:
X-RAY DIFFRACTION

The reported resolution of this entry is 2.00 Å.

Percentile scores (ranging between 0-100) for global validation metrics of the entry are shown in the following graphic. The table shows the number of entries on which the scores are based.

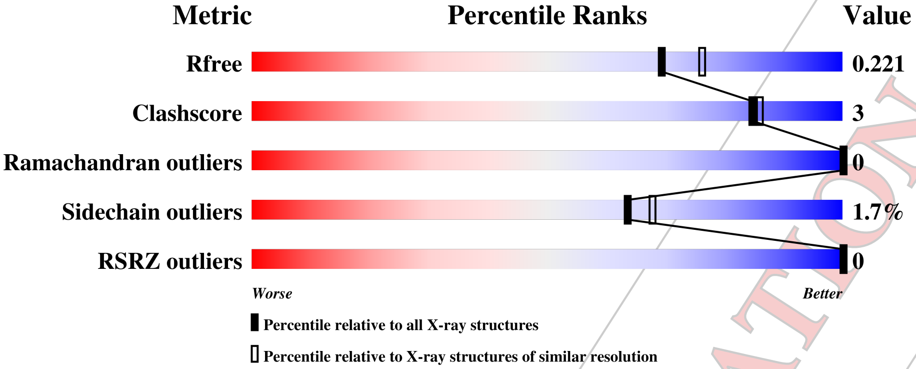

Metric	Whole archive (#Entries)	Similar resolution (#Entries, resolution range(Å))
R_{free}	111664	7193 (2.00-2.00)
Clashscore	122126	8267 (2.00-2.00)
Ramachandran outliers	120053	8166 (2.00-2.00)
Sidechain outliers	120020	8165 (2.00-2.00)
RSRZ outliers	108989	7011 (2.00-2.00)

The table below summarises the geometric issues observed across the polymeric chains and their fit to the electron density. The red, orange, yellow and green segments on the lower bar indicate the fraction of residues that contain outliers for >=3, 2, 1 and 0 types of geometric quality criteria respectively. A grey segment represents the fraction of residues that are not modelled. The numeric value for each fraction is indicated below the corresponding segment, with a dot representing fractions <=5% The upper red bar (where present) indicates the fraction of residues that have poor fit to the electron density. The numeric value is given above the bar.

Mol	Chain	Length	Quality of chain
1	A	271	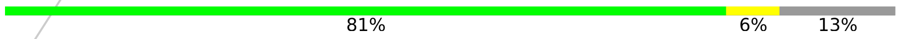

CONFIDENTIAL VALIDATION REPORT

2 Entry composition (i)

There are 5 unique types of molecules in this entry. The entry contains 1980 atoms, of which 0 are hydrogens and 0 are deuteriums.

In the tables below, the ZeroOcc column contains the number of atoms modelled with zero occupancy, the AltConf column contains the number of residues with at least one atom in alternate conformation and the Trace column contains the number of residues modelled with at most 2 atoms.

- Molecule 1 is a protein called Anti-CD33 conditional scFv.

Mol	Chain	Residues	Atoms					ZeroOcc	AltConf	Trace
			Total	C	N	O	S			
1	A	236	1769	1108	306	349	6	0	2	0

- Molecule 2 is PHOSPHATE ION (three-letter code: PO4) (formula: O_4P).

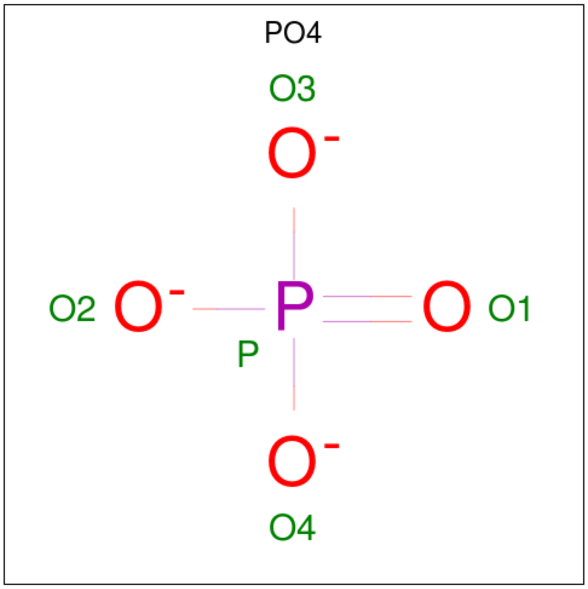

Mol	Chain	Residues	Atoms			ZeroOcc	AltConf
			Total	O	P		
2	A	1	5	4	1	0	0

- Molecule 3 is GLYCEROL (three-letter code: GOL) (formula: $C_3H_8O_3$).

CONFIDENTIAL VALIDATION REPORT

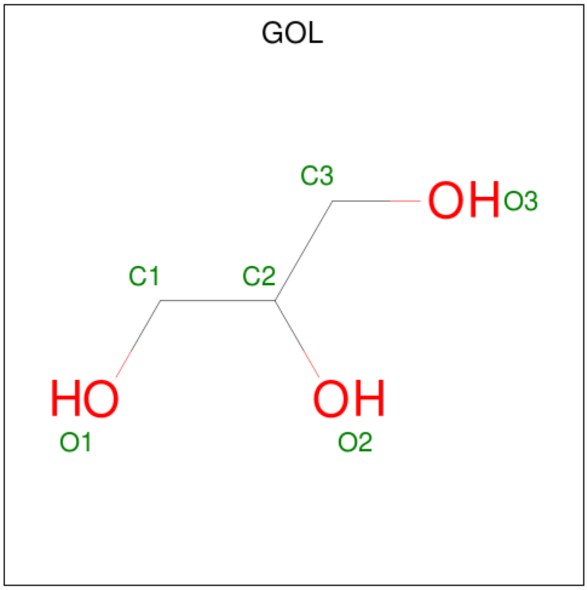

Mol	Chain	Residues	Atoms	ZeroOcc	AltConf
3	A	1	Total C O<br>6 3 3	0	0
3	A	1	Total C O<br>6 3 3	0	0
3	A	1	Total C O<br>6 3 3	0	0
3	A	1	Total C O<br>6 3 3	0	0

- Molecule 4 is METHOTREXATE (three-letter code: MTX) (formula: $C_{20}H_{22}N_8O_5$) (labeled as "Ligand of Interest" by author).

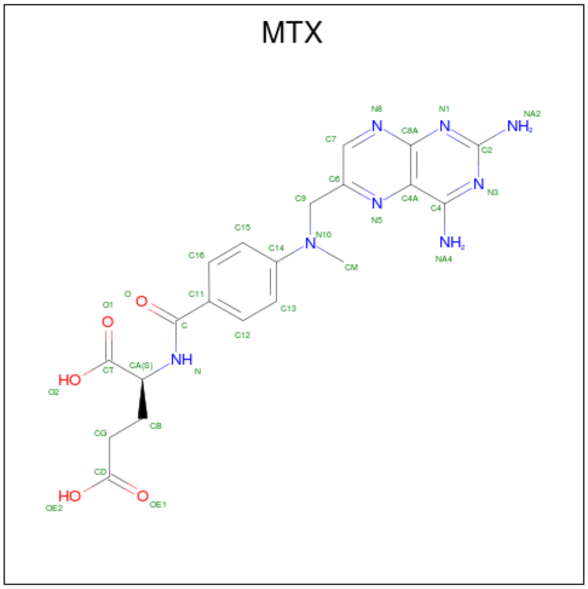

Mol	Chain	Residues	Atoms				ZeroOcc	AltConf
			Total	C	N	O		
4	A	1	33	20	8	5	0	0

- Molecule 5 is water.

Mol	Chain	Residues	Atoms		ZeroOcc	AltConf
			Total	O		
5	A	149	149	149	0	0

CONFIDENTIAL VALIDATION REPORT

3 Residue-property plots (i)

These plots are drawn for all protein, RNA and DNA chains in the entry. The first graphic for a chain summarises the proportions of the various outlier classes displayed in the second graphic. The second graphic shows the sequence view annotated by issues in geometry and electron density. Residues are color-coded according to the number of geometric quality criteria for which they contain at least one outlier: green = 0, yellow = 1, orange = 2 and red = 3 or more. A red dot above a residue indicates a poor fit to the electron density (RSRZ > 2). Stretches of 2 or more consecutive residues without any outlier are shown as a green connector. Residues present in the sample, but not in the model, are shown in grey.

- Molecule 1: Anti-CD33 conditional scFv

Chain A:
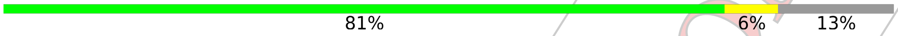

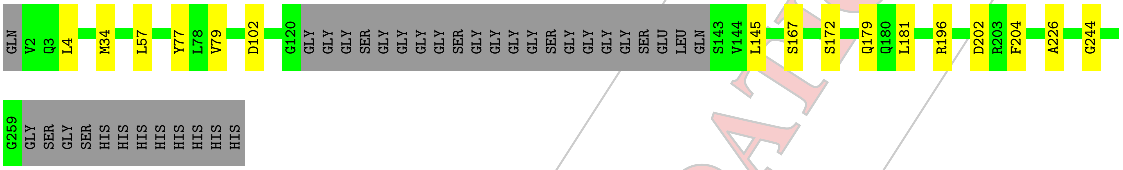

CONFIDENTIAL VALIDATION REPORT

4 Data and refinement statistics (i)

Property	Value	Source
Space group	I 21 3	Depositor
Cell constants<br>a, b, c, α, β, γ	118.99Å 118.99Å 118.99Å<br>90.00° 90.00° 90.00°	Depositor
Resolution (Å)	59.50 – 2.00<br>84.14 – 1.87	Depositor<br>EDS
% Data completeness<br>(in resolution range)	99.3 (59.50-2.00)<br>91.9 (84.14-1.87)	Depositor<br>EDS
R_{merge}	(Not available)	Depositor
R_{sym}	(Not available)	Depositor
$< I/\sigma(I) >$ [1]	0.88 (at 1.87Å)	Xtriage
Refinement program	PHENIX 1.14_3260	Depositor
R, R_{free}	0.189 , 0.221<br>0.189 , 0.221	Depositor<br>DCC
R_{free} test set	904 reflections (3.95%)	wwPDB-VP
Wilson B-factor (Å^2)	30.6	Xtriage
Anisotropy	0.000	Xtriage
Bulk solvent k_{sol}(e/Å^3), B_{sol}(Å^2)	0.35 , 49.1	EDS
L-test for twinning[2]	$< \|L\| > = 0.50$, $< L^2 > = 0.33$	Xtriage
Estimated twinning fraction	0.031 for -l,-k,-h	Xtriage
F_o,F_c correlation	0.96	EDS
Total number of atoms	1980	wwPDB-VP
Average B, all atoms (Å^2)	40.0	wwPDB-VP

Xtriage's analysis on translational NCS is as follows: *The largest off-origin peak in the Patterson function is 5.02% of the height of the origin peak. No significant pseudotranslation is detected.*

[1] Intensities estimated from amplitudes.

[2] Theoretical values of $< |L| >$, $< L^2 >$ for acentric reflections are 0.5, 0.333 respectively for untwinned datasets, and 0.375, 0.2 for perfectly twinned datasets.

CONFIDENTIAL VALIDATION REPORT

5 Model quality (i)

5.1 Standard geometry (i)

Bond lengths and bond angles in the following residue types are not validated in this section: GOL, MTX, PO4

The Z score for a bond length (or angle) is the number of standard deviations the observed value is removed from the expected value. A bond length (or angle) with $|Z| > 5$ is considered an outlier worth inspection. RMSZ is the root-mean-square of all Z scores of the bond lengths (or angles).

Mol	Chain	Bond lengths		Bond angles	
		RMSZ	#$\|Z\|$ >5	RMSZ	#$\|Z\|$ >5
1	A	0.25	0/1814	0.45	0/2463

There are no bond length outliers.

There are no bond angle outliers.

There are no chirality outliers.

There are no planarity outliers.

5.2 Too-close contacts (i)

In the following table, the Non-H and H(model) columns list the number of non-hydrogen atoms and hydrogen atoms in the chain respectively. The H(added) column lists the number of hydrogen atoms added and optimized by MolProbity. The Clashes column lists the number of clashes within the asymmetric unit, whereas Symm-Clashes lists symmetry related clashes.

Mol	Chain	Non-H	H(model)	H(added)	Clashes	Symm-Clashes
1	A	1769	0	1672	8	0
2	A	5	0	0	0	0
3	A	24	0	32	1	0
4	A	33	0	20	2	0
5	A	149	0	0	0	0
All	All	1980	0	1724	9	0

The all-atom clashscore is defined as the number of clashes found per 1000 atoms (including hydrogen atoms). The all-atom clashscore for this structure is 3.

All (9) close contacts within the same asymmetric unit are listed below, sorted by their clash magnitude.

CONFIDENTIAL VALIDATION REPORT

Atom-1	Atom-2	Interatomic distance (Å)	Clash overlap (Å)
1:A:181:LEU:HD23	1:A:226:ALA:HB2	1.85	0.58
1:A:145:LEU:HB2	1:A:244:GLY:HA2	1.91	0.52
1:A:167:SER:O	1:A:172:SER:OG	2.19	0.49
1:A:179:GLN:HE22	3:A:302:GOL:H11	1.77	0.49
1:A:4:LEU:HD21	4:A:306:MTX:H91	1.97	0.45
1:A:34:MET:HG3	1:A:79:VAL:HG21	1.98	0.45
1:A:196:ARG:HD3	1:A:204:PHE:O	2.18	0.44
4:A:306:MTX:H13	4:A:306:MTX:HM1	1.57	0.43
1:A:57:LEU:HD12	1:A:57:LEU:HA	1.92	0.42

There are no symmetry-related clashes.

5.3 Torsion angles (i)

5.3.1 Protein backbone (i)

In the following table, the Percentiles column shows the percent Ramachandran outliers of the chain as a percentile score with respect to all X-ray entries followed by that with respect to entries of similar resolution.

The Analysed column shows the number of residues for which the backbone conformation was analysed, and the total number of residues.

Mol	Chain	Analysed	Favoured	Allowed	Outliers	Percentiles	
1	A	234/271 (86%)	230 (98%)	4 (2%)	0	100	100

There are no Ramachandran outliers to report.

5.3.2 Protein sidechains (i)

In the following table, the Percentiles column shows the percent sidechain outliers of the chain as a percentile score with respect to all X-ray entries followed by that with respect to entries of similar resolution.

The Analysed column shows the number of residues for which the sidechain conformation was analysed, and the total number of residues.

Mol	Chain	Analysed	Rotameric	Outliers	Percentiles	
1	A	182/205 (89%)	179 (98%)	3 (2%)	65	70

All (3) residues with a non-rotameric sidechain are listed below:

CONFIDENTIAL VALIDATION REPORT

Mol	Chain	Res	Type
1	A	77	TYR
1	A	102	ASP
1	A	202	ASP

Some sidechains can be flipped to improve hydrogen bonding and reduce clashes. There are no such sidechains identified.

5.3.3 RNA (i)

There are no RNA molecules in this entry.

5.4 Non-standard residues in protein, DNA, RNA chains (i)

There are no non-standard protein/DNA/RNA residues in this entry.

5.5 Carbohydrates (i)

There are no carbohydrates in this entry.

5.6 Ligand geometry (i)

6 ligands are modelled in this entry.

In the following table, the Counts columns list the number of bonds (or angles) for which Mogul statistics could be retrieved, the number of bonds (or angles) that are observed in the model and the number of bonds (or angles) that are defined in the Chemical Component Dictionary. The Link column lists molecule types, if any, to which the group is linked. The Z score for a bond length (or angle) is the number of standard deviations the observed value is removed from the expected value. A bond length (or angle) with $|Z| > 2$ is considered an outlier worth inspection. RMSZ is the root-mean-square of all Z scores of the bond lengths (or angles).

Mol	Type	Chain	Res	Link	Bond lengths			Bond angles		
					Counts	RMSZ	$\#\|Z\| > 2$	Counts	RMSZ	$\#\|Z\| > 2$
2	PO4	A	301	-	4,4,4	0.92	0	6,6,6	0.42	0
3	GOL	A	302	-	5,5,5	0.89	0	5,5,5	0.99	0
3	GOL	A	303	-	5,5,5	0.89	0	5,5,5	0.99	0
3	GOL	A	304	-	5,5,5	0.89	0	5,5,5	1.01	0
3	GOL	A	305	-	5,5,5	0.90	0	5,5,5	1.01	0
4	MTX	A	306	-	29,35,35	2.06	4 (13%)	37,49,49	1.86	9 (24%)

In the following table, the Chirals column lists the number of chiral outliers, the number of chiral

CONFIDENTIAL VALIDATION REPORT

centers analysed, the number of these observed in the model and the number defined in the Chemical Component Dictionary. Similar counts are reported in the Torsion and Rings columns. '-' means no outliers of that kind were identified.

Mol	Type	Chain	Res	Link	Chirals	Torsions	Rings
3	GOL	A	302	-	-	0/4/4/4	-
3	GOL	A	303	-	-	0/4/4/4	-
3	GOL	A	304	-	-	0/4/4/4	-
3	GOL	A	305	-	-	0/4/4/4	-
4	MTX	A	306	-	-	2/19/25/25	0/3/3/3

All (4) bond length outliers are listed below:

Mol	Chain	Res	Type	Atoms	Z	Observed(Å)	Ideal(Å)
4	A	306	MTX	O-C	8.86	1.41	1.23
4	A	306	MTX	C7-N8	4.11	1.38	1.31
4	A	306	MTX	C6-N5	3.29	1.38	1.32
4	A	306	MTX	C4-N3	2.51	1.38	1.33

All (9) bond angle outliers are listed below:

Mol	Chain	Res	Type	Atoms	Z	Observed(^{o})	Ideal(^{o})
4	A	306	MTX	N1-C2-N3	-5.27	120.13	127.25
4	A	306	MTX	C2-N1-C8A	4.15	120.09	115.36
4	A	306	MTX	N8-C8A-N1	3.68	120.19	115.83
4	A	306	MTX	C13-C14-N10	-3.15	117.07	121.62
4	A	306	MTX	C6-C7-N8	-2.93	120.25	123.13
4	A	306	MTX	C4A-C4-N3	-2.20	119.52	121.02
4	A	306	MTX	C7-C6-N5	-2.17	119.43	120.85
4	A	306	MTX	CB-CA-CT	-2.12	109.12	112.18
4	A	306	MTX	CM-N10-C14	-2.10	115.98	119.60

There are no chirality outliers.

All (2) torsion outliers are listed below:

Mol	Chain	Res	Type	Atoms
4	A	306	MTX	C6-C9-N10-C14
4	A	306	MTX	CA-CB-CG-CD

There are no ring outliers.

2 monomers are involved in 3 short contacts:

CONFIDENTIAL VALIDATION REPORT

Mol	Chain	Res	Type	Clashes	Symm-Clashes
3	A	302	GOL	1	0
4	A	306	MTX	2	0

The following is a two-dimensional graphical depiction of Mogul quality analysis of bond lengths, bond angles, torsion angles, and ring geometry for all instances of the Ligand of Interest. In addition, ligands with molecular weight > 250 and outliers as shown on the validation Tables will also be included. For torsion angles, if less then 5% of the Mogul distribution of torsion angles is within 10 degrees of the torsion angle in question, then that torsion angle is considered an outlier. Any bond that is central to one or more torsion angles identified as an outlier by Mogul will be highlighted in the graph. For rings, the root-mean-square deviation (RMSD) between the ring in question and similar rings identified by Mogul is calculated over all ring torsion angles. If the average RMSD is greater than 60 degrees and the minimal RMSD between the ring in question and any Mogul-identified rings is also greater than 60 degrees, then that ring is considered an outlier. The outliers are highlighted in purple. The color gray indicates Mogul did not find sufficient equivalents in the CSD to analyse the geometry.

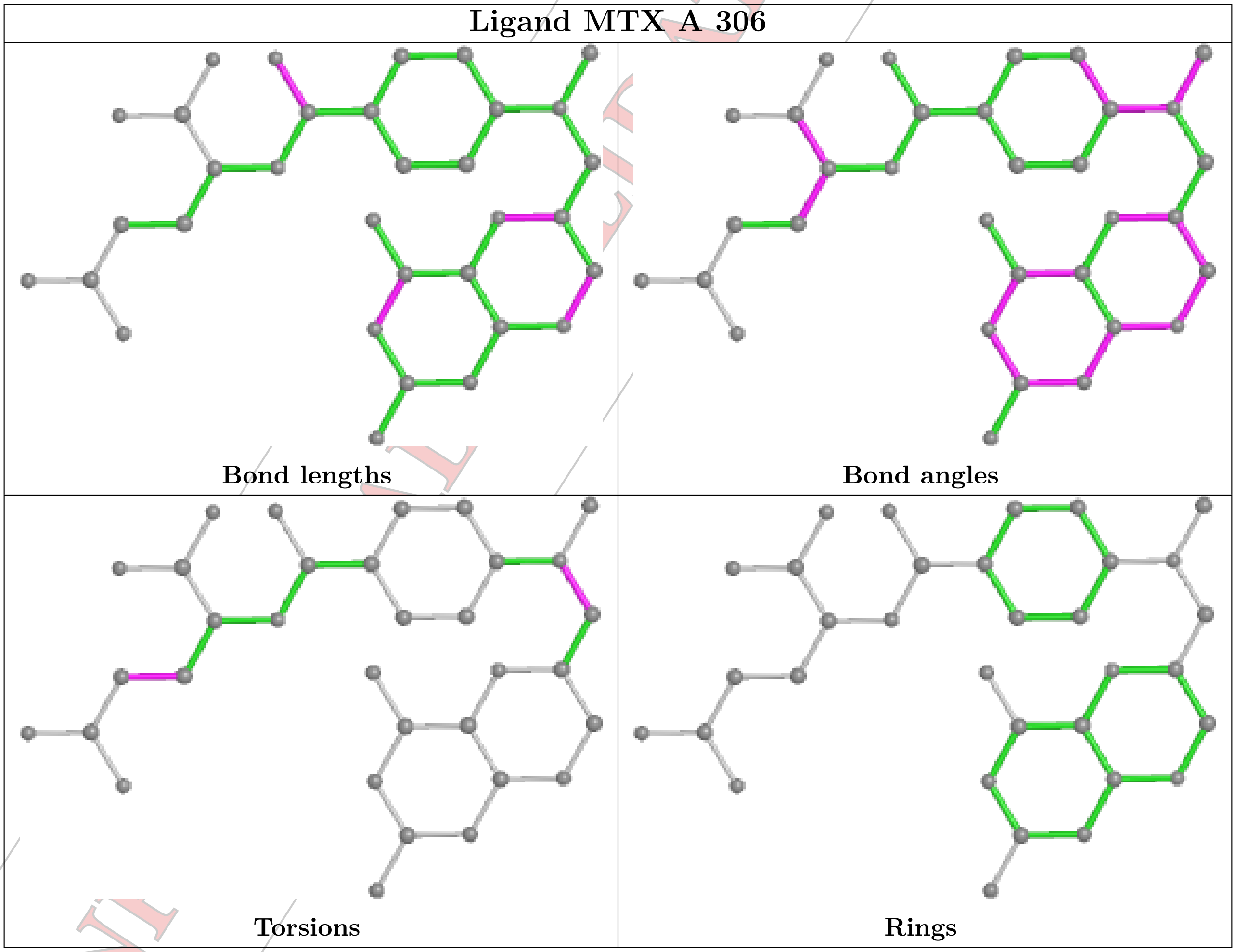

5.7 Other polymers (i)

There are no such residues in this entry.

5.8 Polymer linkage issues (i)

There are no chain breaks in this entry.

CONFIDENTIAL VALIDATION REPORT

6 Fit of model and data (i)

6.1 Protein, DNA and RNA chains (i)

In the following table, the column labelled '#RSRZ> 2' contains the number (and percentage) of RSRZ outliers, followed by percent RSRZ outliers for the chain as percentile scores relative to all X-ray entries and entries of similar resolution. The OWAB column contains the minimum, median, 95th percentile and maximum values of the occupancy-weighted average B-factor per residue. The column labelled 'Q< 0.9' lists the number of (and percentage) of residues with an average occupancy less than 0.9.

Mol	Chain	Analysed	<RSRZ>	#RSRZ>2			OWAB(Å^2)	Q<0.9
1	A	236/271 (87%)	-0.22	0	100	100	27, 38, 55, 68	0

There are no RSRZ outliers to report.

6.2 Non-standard residues in protein, DNA, RNA chains (i)

There are no non-standard protein/DNA/RNA residues in this entry.

6.3 Carbohydrates (i)

There are no carbohydrates in this entry.

6.4 Ligands (i)

In the following table, the Atoms column lists the number of modelled atoms in the group and the number defined in the chemical component dictionary. The B-factors column lists the minimum, median, 95th percentile and maximum values of B factors of atoms in the group. The column labelled 'Q< 0.9' lists the number of atoms with occupancy less than 0.9.

Mol	Type	Chain	Res	Atoms	RSCC	RSR	B-factors(Å^2)	Q<0.9
3	GOL	A	305	6/6	0.75	0.26	51,63,65,70	0
2	PO4	A	301	5/5	0.77	0.31	85,91,93,112	0
3	GOL	A	303	6/6	0.80	0.54	57,61,80,84	0
3	GOL	A	302	6/6	0.80	0.19	52,61,68,71	0
3	GOL	A	304	6/6	0.89	0.17	55,58,60,61	0
4	MTX	A	306	33/33	0.93	0.12	33,43,63,66	0

The following is a graphical depiction of the model fit to experimental electron density of all instances of the Ligand of Interest. In addition, ligands with molecular weight > 250 and outliers as shown on the geometry validation Tables will also be included. Each fit is shown from different

CONFIDENTIAL VALIDATION REPORT

orientation to approximate a three-dimensional view.

Electron density around MTX A 306:

$2mF_o$-DF_c (at 0.7 rmsd) in gray
mF_o-DF_c (at 3 rmsd) in purple (negative)
and green (positive)

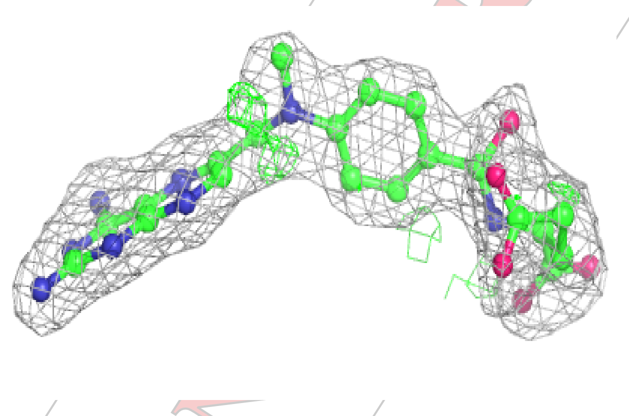

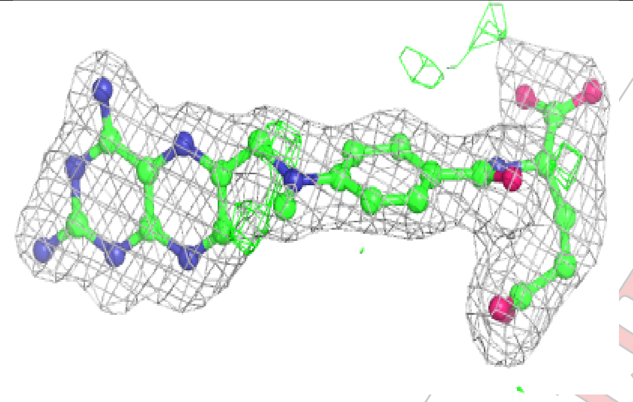

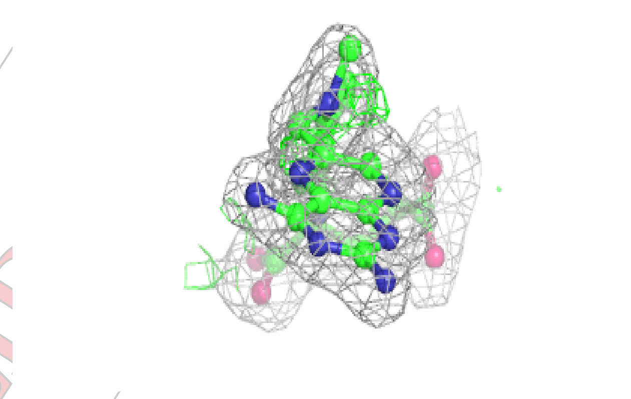

6.5 Other polymers (i)

There are no such residues in this entry.

CONFIDENTIAL VALIDATION REPORT

CONFIDENTIAL VALIDATION REPORT

Full wwPDB X-ray Structure Validation Report (i)

Nov 1, 2019 – 02:06 PM EDT

PDB ID	:	6UUP
Title	:	Structure of anti-hCD33 conditional scFv
Deposited on	:	2019-10-31
Resolution	:	2.20 Å(reported)

This is a Full wwPDB X-ray Structure Validation Report.

This report is produced by the wwPDB biocuration pipeline after annotation of the structure.

We welcome your comments at *validation@mail.wwpdb.org*
A user guide is available at
https://www.wwpdb.org/validation/2017/XrayValidationReportHelp
with specific help available everywhere you see the (i) symbol.

The following versions of software and data (see references (i)) were used in the production of this report:

MolProbity	:	4.02b-467
Mogul	:	1.8.0 (224370), CSD as540be (2019)
Xtriage (Phenix)	:	1.13
EDS	:	2.5
Percentile statistics	:	20171227.v01 (using entries in the PDB archive December 27th 2017)
Refmac	:	5.8.0158
CCP4	:	7.0 (Gargrove)
Ideal geometry (proteins)	:	Engh & Huber (2001)
Ideal geometry (DNA, RNA)	:	Parkinson et al. (1996)
Validation Pipeline (wwPDB-VP)	:	2.5

1 Overall quality at a glance (i)

The following experimental techniques were used to determine the structure:
X-RAY DIFFRACTION

The reported resolution of this entry is 2.20 Å.

Percentile scores (ranging between 0-100) for global validation metrics of the entry are shown in the following graphic. The table shows the number of entries on which the scores are based.

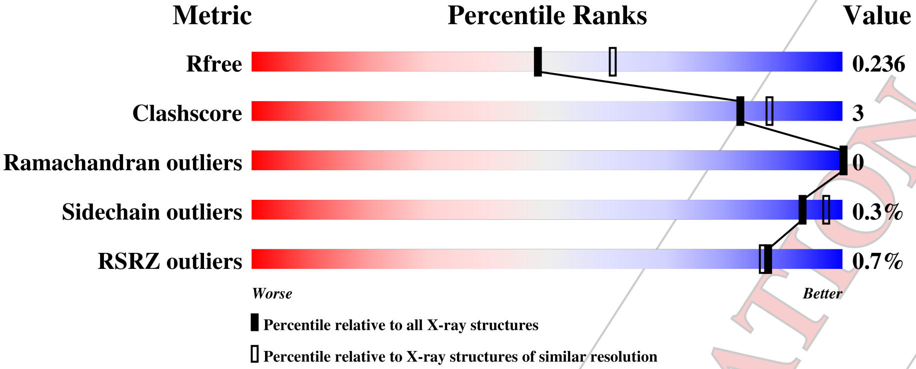

Metric	Whole archive (#Entries)	Similar resolution (#Entries, resolution range(Å))
R_{free}	111664	4343 (2.20-2.20)
Clashscore	122126	5027 (2.20-2.20)
Ramachandran outliers	120053	4952 (2.20-2.20)
Sidechain outliers	120020	4953 (2.20-2.20)
RSRZ outliers	108989	4245 (2.20-2.20)

The table below summarises the geometric issues observed across the polymeric chains and their fit to the electron density. The red, orange, yellow and green segments on the lower bar indicate the fraction of residues that contain outliers for >=3, 2, 1 and 0 types of geometric quality criteria. A grey segment represents the fraction of residues that are not modelled. The numeric value for each fraction is indicated below the corresponding segment, with a dot representing fractions <=5% The upper red bar (where present) indicates the fraction of residues that have poor fit to the electron density. The numeric value is given above the bar.

Mol	Chain	Length	Quality of chain
1	A	271	79% • 16%
1	B	271	% 78% 6% 16%
1	C	271	79% 5% 16%
1	D	271	% 79% 5% 15%

CONFIDENTIAL VALIDATION REPORT

2 Entry composition (i)

There are 6 unique types of molecules in this entry. The entry contains 7645 atoms, of which 0 are hydrogens and 0 are deuteriums.

In the tables below, the ZeroOcc column contains the number of atoms modelled with zero occupancy, the AltConf column contains the number of residues with at least one atom in alternate conformation and the Trace column contains the number of residues modelled with at most 2 atoms.

- Molecule 1 is a protein called Anti-CD33 conditional scFv.

Mol	Chain	Residues	Atoms	ZeroOcc	AltConf	Trace
1	A	227	Total C N O S<br>1679 1051 289 333 6	0	0	0
1	B	228	Total C N O S<br>1696 1063 293 334 6	0	0	0
1	C	227	Total C N O S<br>1695 1058 297 334 6	0	0	0
1	D	229	Total C N O S<br>1702 1066 296 334 6	0	0	0

- Molecule 2 is GLYCEROL (three-letter code: GOL) (formula: $C_3H_8O_3$).

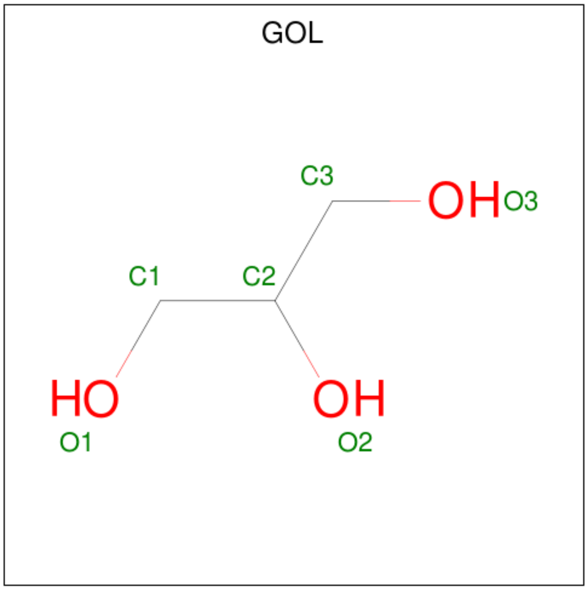

Mol	Chain	Residues	Atoms	ZeroOcc	AltConf
2	A	1	Total C O<br>6 3 3	0	0
2	A	1	Total C O<br>6 3 3	0	0

Continued on next page...

CONFIDENTIAL VALIDATION REPORT

Continued from previous page...

Mol	Chain	Residues	Atoms	ZeroOcc	AltConf
2	A	1	Total C O<br>6 3 3	0	0
2	A	1	Total C O<br>6 3 3	0	0
2	A	1	Total C O<br>6 3 3	0	0
2	A	1	Total C O<br>6 3 3	0	0
2	A	1	Total C O<br>6 3 3	0	0
2	A	1	Total C O<br>6 3 3	0	0
2	B	1	Total C O<br>6 3 3	0	0
2	C	1	Total C O<br>6 3 3	0	0
2	C	1	Total C O<br>6 3 3	0	0
2	C	1	Total C O<br>6 3 3	0	0
2	C	1	Total C O<br>6 3 3	0	0
2	C	1	Total C O<br>6 3 3	0	0
2	C	1	Total C O<br>6 3 3	0	0
2	C	1	Total C O<br>6 3 3	0	0
2	D	1	Total C O<br>6 3 3	0	0
2	D	1	Total C O<br>6 3 3	0	0
2	D	1	Total C O<br>6 3 3	0	0

- Molecule 3 is DI(HYDROXYETHYL)ETHER (three-letter code: PEG) (formula: $C_4H_{10}O_3$).

CONFIDENTIAL VALIDATION REPORT

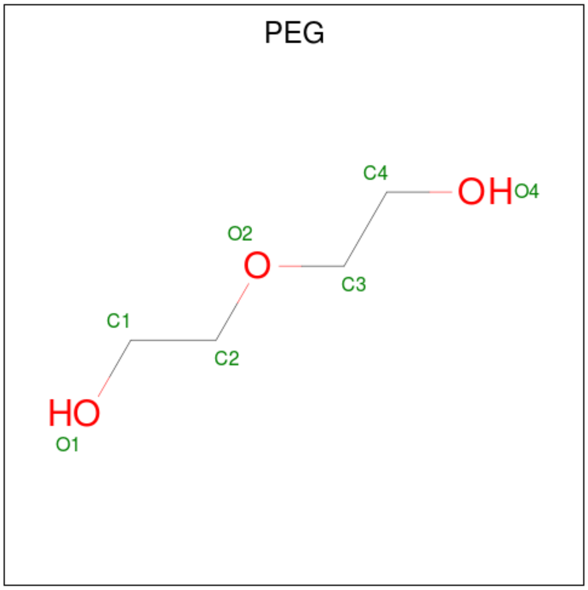

Mol	Chain	Residues	Atoms	ZeroOcc	AltConf
3	A	1	Total C O<br>7 4 3	0	0
3	B	1	Total C O<br>7 4 3	0	0
3	C	1	Total C O<br>7 4 3	0	0
3	D	1	Total C O<br>7 4 3	0	0

- Molecule 4 is POTASSIUM ION (three-letter code: K) (formula: K).

Mol	Chain	Residues	Atoms	ZeroOcc	AltConf
4	B	2	Total K<br>2 2	0	0
4	A	2	Total K<br>2 2	0	0
4	D	2	Total K<br>2 2	0	0
4	C	2	Total K<br>2 2	0	0

- Molecule 5 is 2-AMINO-2-HYDROXYMETHYL-PROPANE-1,3-DIOL (three-letter code: TRS) (formula: $C_4H_{12}NO_3$).

CONFIDENTIAL VALIDATION REPORT

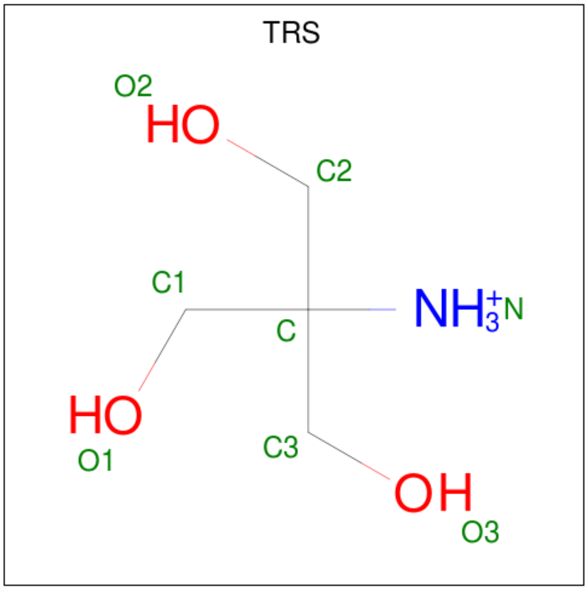

Mol	Chain	Residues	Atoms	ZeroOcc	AltConf
5	D	1	Total C N O<br>8 4 1 3	0	0
5	D	1	Total C N O<br>8 4 1 3	0	0

- Molecule 6 is water.

Mol	Chain	Residues	Atoms	ZeroOcc	AltConf
6	A	182	Total O<br>182 182	0	0
6	B	170	Total O<br>170 170	0	0
6	C	170	Total O<br>170 170	0	0
6	D	185	Total O<br>185 185	0	0

CONFIDENTIAL VALIDATION REPORT

3 Residue-property plots ⓘ

These plots are drawn for all protein, RNA and DNA chains in the entry. The first graphic for a chain summarises the proportions of the various outlier classes displayed in the second graphic. The second graphic shows the sequence view annotated by issues in geometryand electron density. Residues are color-coded according to the number of geometric quality criteria for which they contain at least one outlier: green = 0, yellow = 1, orange = 2 and red = 3 or more. A red dot above a residue indicates a poor fit to the electron density (RSRZ > 2). Stretches of 2 or more consecutive residues without any outlier are shown as a green connector. Residues present in the sample, but not in the model, are shown in grey.

- Molecule 1: Anti-CD33 conditional scFv

Chain A:

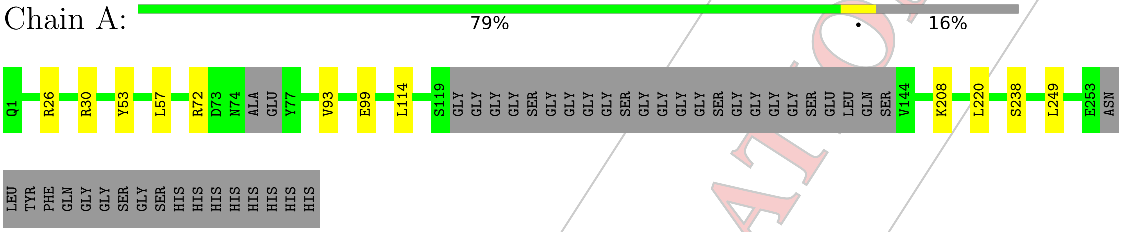

- Molecule 1: Anti-CD33 conditional scFv

Chain B:

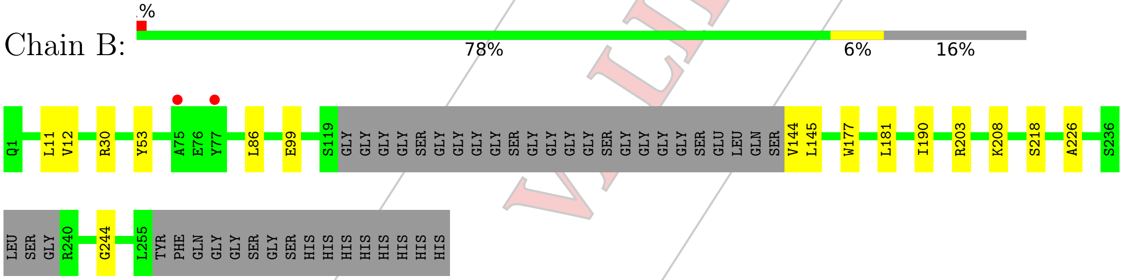

- Molecule 1: Anti-CD33 conditional scFv

Chain C:

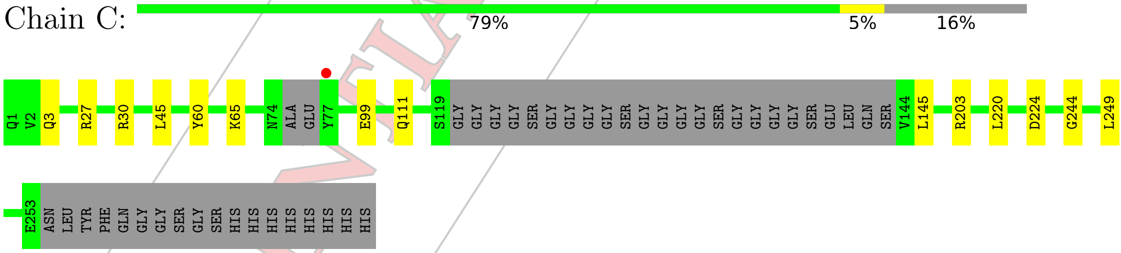

- Molecule 1: Anti-CD33 conditional scFv

Chain D:

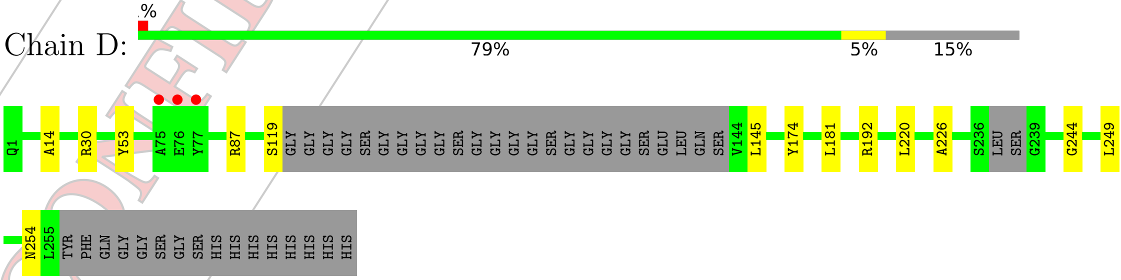

CONFIDENTIAL VALIDATION REPORT

4 Data and refinement statistics (i)

Property	Value	Source
Space group	P 1 21 1	Depositor
Cell constants<br>a, b, c, α, β, γ	81.23Å 104.64Å 87.92Å<br>90.00° 113.57° 90.00°	Depositor
Resolution (Å)	46.24 – 2.20<br>46.24 – 2.02	Depositor<br>EDS
% Data completeness<br>(in resolution range)	99.9 (46.24-2.20)<br>86.6 (46.24-2.02)	Depositor<br>EDS
R_{merge}	0.19	Depositor
R_{sym}	(Not available)	Depositor
$< I/\sigma(I) >$ [1]	0.90 (at 2.01Å)	Xtriage
Refinement program	PHENIX 1.11.1_2575	Depositor
R, R_{free}	0.197 , 0.237<br>0.198 , 0.236	Depositor<br>DCC
R_{free} test set	1984 reflections (2.25%)	wwPDB-VP
Wilson B-factor (Å^2)	22.2	Xtriage
Anisotropy	0.333	Xtriage
Bulk solvent k_{sol}(e/Å^3), B_{sol}(Å^2)	0.35 , 49.1	EDS
L-test for twinning[2]	$< \|L\| > = 0.49$, $< L^2 > = 0.32$	Xtriage
Estimated twinning fraction	No twinning to report.	Xtriage
F_o,F_c correlation	0.94	EDS
Total number of atoms	7645	wwPDB-VP
Average B, all atoms (Å^2)	33.0	wwPDB-VP

Xtriage's analysis on translational NCS is as follows: *The largest off-origin peak in the Patterson function is 9.98% of the height of the origin peak. No significant pseudotranslation is detected.*

[1] Intensities estimated from amplitudes.
[2] Theoretical values of $< |L| >$, $< L^2 >$ for acentric reflections are 0.5, 0.333 respectively for untwinned datasets, and 0.375, 0.2 for perfectly twinned datasets.

CONFIDENTIAL VALIDATION REPORT

5 Model quality ⓘ

5.1 Standard geometry ⓘ

Bond lengths and bond angles in the following residue types are not validated in this section: GOL, K, PEG, TRS

The Z score for a bond length (or angle) is the number of standard deviations the observed value is removed from the expected value. A bond length (or angle) with $|Z| > 5$ is considered an outlier worth inspection. RMSZ is the root-mean-square of all Z scores of the bond lengths (or angles).

Mol	Chain	Bond lengths		Bond angles	
		RMSZ	$\#\|Z\| > 5$	RMSZ	$\#\|Z\| > 5$
1	A	0.26	0/1715	0.47	0/2330
1	B	0.26	0/1732	0.45	0/2351
1	C	0.26	0/1731	0.46	0/2349
1	D	0.26	0/1738	0.46	0/2359
All	All	0.26	0/6916	0.46	0/9389

There are no bond length outliers.

There are no bond angle outliers.

There are no chirality outliers.

There are no planarity outliers.

5.2 Too-close contacts ⓘ

In the following table, the Non-H and H(model) columns list the number of non-hydrogen atoms and hydrogen atoms in the chain respectively. The H(added) column lists the number of hydrogen atoms added and optimized by MolProbity. The Clashes column lists the number of clashes within the asymmetric unit, whereas Symm-Clashes lists symmetry related clashes.

Mol	Chain	Non-H	H(model)	H(added)	Clashes	Symm-Clashes
1	A	1679	0	1569	10	0
1	B	1696	0	1603	11	0
1	C	1695	0	1599	10	0
1	D	1702	0	1608	11	0
2	A	48	0	64	1	0
2	B	6	0	8	1	0
2	C	42	0	56	1	0
2	D	18	0	24	2	0
3	A	7	0	10	1	0

Continued on next page...

CONFIDENTIAL VALIDATION REPORT

Continued from previous page...

Mol	Chain	Non-H	H(model)	H(added)	Clashes	Symm-Clashes
3	B	7	0	10	1	0
3	C	7	0	10	0	0
3	D	7	0	10	0	0
4	A	2	0	0	0	0
4	B	2	0	0	0	0
4	C	2	0	0	0	0
4	D	2	0	0	0	0
5	D	16	0	24	0	0
6	A	182	0	0	1	0
6	B	170	0	0	0	0
6	C	170	0	0	0	0
6	D	185	0	0	0	0
All	All	7645	0	6595	36	0

The all-atom clashscore is defined as the number of clashes found per 1000 atoms (including hydrogen atoms). The all-atom clashscore for this structure is 3.

All (36) close contacts within the same asymmetric unit are listed below, sorted by their clash magnitude.

Atom-1	Atom-2	Interatomic distance (Å)	Clash overlap (Å)
1:A:26:ARG:HH12	2:A:301:GOL:H31	1.60	0.66
1:D:87:ARG:HH22	2:D:504:GOL:H11	1.64	0.62
1:D:87:ARG:HH12	2:D:504:GOL:H2	1.67	0.57
1:D:145:LEU:HB2	1:D:244:GLY:HA2	1.87	0.56
1:A:208:LYS:NZ	3:A:309:PEG:O4	2.38	0.55
1:C:3:GLN:HA	2:C:301:GOL:H11	1.88	0.54
1:A:93:VAL:HG22	1:A:114:LEU:HD13	1.90	0.53
1:A:53:TYR:OH	1:B:99:GLU:OE1	2.28	0.52
1:C:145:LEU:HB2	1:C:244:GLY:HA2	1.93	0.50
1:C:220:LEU:HD11	1:C:249:LEU:HD21	1.93	0.50
1:A:93:VAL:CG2	1:A:114:LEU:HD13	2.41	0.50
1:C:99:GLU:OE1	1:D:53:TYR:OH	2.30	0.50
1:D:14:ALA:HB2	1:D:119:SER:HA	1.93	0.50
1:B:145:LEU:HB2	1:B:244:GLY:HA2	1.95	0.49
1:B:208:LYS:NZ	3:B:302:PEG:O1	2.44	0.49
1:C:27:ARG:NH2	1:D:192:ARG:HH12	2.12	0.47
1:C:203:ARG:NH1	1:C:224:ASP:OD2	2.42	0.47
1:D:220:LEU:HD11	1:D:249:LEU:HD21	1.96	0.47
1:D:174:TYR:CG	1:D:192:ARG:HG2	2.50	0.47
1:A:220:LEU:HD11	1:A:249:LEU:HD21	1.98	0.46
1:A:99:GLU:OE1	1:B:53:TYR:OH	2.34	0.46

Continued on next page...

CONFIDENTIAL VALIDATION REPORT

Continued from previous page...

Atom-1	Atom-2	Interatomic distance (Å)	Clash overlap (Å)
1:C:30:ARG:HA	1:D:30:ARG:HA	1.98	0.46
1:B:11:LEU:HD23	1:B:12:VAL:N	2.32	0.45
1:B:12:VAL:HG11	1:B:86:LEU:HD13	1.99	0.44
1:C:3:GLN:HE22	1:C:111:GLN:HE22	1.65	0.44
1:A:30:ARG:HA	1:B:30:ARG:HA	1.99	0.44
1:B:203:ARG:HB3	1:B:218:SER:O	2.18	0.44
1:A:72:ARG:NH2	6:A:414:HOH:O	2.51	0.43
1:B:181:LEU:HD23	1:B:226:ALA:HB2	1.98	0.43
1:B:144:VAL:HG22	1:B:145:LEU:H	1.84	0.42
1:A:57:LEU:HD21	2:B:301:GOL:H2	2.01	0.42
1:C:3:GLN:NE2	1:C:111:GLN:HE22	2.17	0.42
1:B:177:TRP:HB2	1:B:190:ILE:HB	2.01	0.42
1:C:60:TYR:HB2	1:C:65:LYS:HD3	2.01	0.42
1:D:181:LEU:HD23	1:D:226:ALA:HB2	2.02	0.40
1:D:254:ASN:N	1:D:254:ASN:OD1	2.54	0.40

There are no symmetry-related clashes.

5.3 Torsion angles (i)

5.3.1 Protein backbone (i)

In the following table, the Percentiles column shows the percent Ramachandran outliers of the chain as a percentile score with respect to all X-ray entries followed by that with respect to entries of similar resolution.

The Analysed column shows the number of residues for which the backbone conformation was analysed, and the total number of residues.

Mol	Chain	Analysed	Favoured	Allowed	Outliers	Percentiles	
1	A	221/271 (82%)	215 (97%)	6 (3%)	0	100	100
1	B	222/271 (82%)	218 (98%)	4 (2%)	0	100	100
1	C	221/271 (82%)	216 (98%)	5 (2%)	0	100	100
1	D	223/271 (82%)	219 (98%)	4 (2%)	0	100	100
All	All	887/1084 (82%)	868 (98%)	19 (2%)	0	100	100

There are no Ramachandran outliers to report.

CONFIDENTIAL VALIDATION REPORT

5.3.2 Protein sidechains (i)

In the following table, the Percentiles column shows the percent sidechain outliers of the chain as a percentile score with respect to all X-ray entries followed by that with respect to entries of similar resolution.

The Analysed column shows the number of residues for which the sidechain conformation was analysed, and the total number of residues.

Mol	Chain	Analysed	Rotameric	Outliers	Percentiles	
1	A	170/205 (83%)	169 (99%)	1 (1%)	87	94
1	B	173/205 (84%)	173 (100%)	0	100	100
1	C	174/205 (85%)	173 (99%)	1 (1%)	87	94
1	D	173/205 (84%)	173 (100%)	0	100	100
All	All	690/820 (84%)	688 (100%)	2 (0%)	93	97

All (2) residues with a non-rotameric sidechain are listed below:

Mol	Chain	Res	Type
1	A	238	SER
1	C	45	LEU

Some sidechains can be flipped to improve hydrogen bonding and reduce clashes. All (3) such sidechains are listed below:

Mol	Chain	Res	Type
1	A	3	GLN
1	A	111	GLN
1	C	3	GLN

5.3.3 RNA (i)

There are no RNA molecules in this entry.

5.4 Non-standard residues in protein, DNA, RNA chains (i)

There are no non-standard protein/DNA/RNA residues in this entry.

5.5 Carbohydrates (i)

There are no carbohydrates in this entry.

CONFIDENTIAL VALIDATION REPORT

5.6 Ligand geometry (i)

Of 33 ligands modelled in this entry, 8 are monoatomic - leaving 25 for Mogul analysis.

In the following table, the Counts columns list the number of bonds (or angles) for which Mogul statistics could be retrieved, the number of bonds (or angles) that are observed in the model and the number of bonds (or angles) that are defined in the Chemical Component Dictionary. The Link column lists molecule types, if any, to which the group is linked. The Z score for a bond length (or angle) is the number of standard deviations the observed value is removed from the expected value. A bond length (or angle) with $|Z| > 2$ is considered an outlier worth inspection. RMSZ is the root-mean-square of all Z scores of the bond lengths (or angles).

Mol	Type	Chain	Res	Link	Bond lengths			Bond angles		
					Counts	RMSZ	$\#\|Z\| > 2$	Counts	RMSZ	$\#\|Z\| > 2$
2	GOL	A	301	-	5,5,5	0.37	0	5,5,5	0.27	0
2	GOL	A	302	-	5,5,5	0.35	0	5,5,5	0.25	0
2	GOL	A	303	-	5,5,5	0.35	0	5,5,5	0.23	0
2	GOL	A	304	-	5,5,5	0.36	0	5,5,5	0.21	0
2	GOL	A	305	-	5,5,5	0.38	0	5,5,5	0.39	0
2	GOL	A	306	-	5,5,5	0.35	0	5,5,5	0.35	0
2	GOL	A	307	-	5,5,5	0.36	0	5,5,5	0.33	0
2	GOL	A	308	-	5,5,5	0.37	0	5,5,5	0.29	0
3	PEG	A	309	-	6,6,6	0.48	0	5,5,5	0.26	0
2	GOL	B	301	-	5,5,5	0.39	0	5,5,5	0.21	0
3	PEG	B	302	-	6,6,6	0.49	0	5,5,5	0.23	0
2	GOL	C	301	-	5,5,5	0.38	0	5,5,5	0.15	0
2	GOL	C	302	-	5,5,5	0.35	0	5,5,5	0.24	0
2	GOL	C	303	-	5,5,5	0.37	0	5,5,5	0.21	0
2	GOL	C	304	-	5,5,5	0.35	0	5,5,5	0.24	0
2	GOL	C	305	-	5,5,5	0.36	0	5,5,5	0.25	0
2	GOL	C	306	-	5,5,5	0.39	0	5,5,5	0.33	0
2	GOL	C	307	-	5,5,5	0.35	0	5,5,5	0.33	0
3	PEG	C	308	-	6,6,6	0.48	0	5,5,5	0.23	0
5	TRS	D	501	-	7,7,7	0.31	0	9,9,9	0.45	0
5	TRS	D	502	-	7,7,7	0.30	0	9,9,9	0.39	0
2	GOL	D	503	-	5,5,5	0.37	0	5,5,5	0.30	0
2	GOL	D	504	-	5,5,5	0.36	0	5,5,5	0.31	0
2	GOL	D	505	-	5,5,5	0.36	0	5,5,5	0.28	0
3	PEG	D	506	-	6,6,6	0.48	0	5,5,5	0.24	0

In the following table, the Chirals column lists the number of chiral outliers, the number of chiral centers analysed, the number of these observed in the model and the number defined in the Chemical Component Dictionary. Similar counts are reported in the Torsion and Rings columns. '-' means no outliers of that kind were identified.

CONFIDENTIAL VALIDATION REPORT

Mol	Type	Chain	Res	Link	Chirals	Torsions	Rings
2	GOL	A	301	-	-	2/4/4/4	-
2	GOL	A	302	-	-	2/4/4/4	-
2	GOL	A	303	-	-	2/4/4/4	-
2	GOL	A	304	-	-	2/4/4/4	-
2	GOL	A	305	-	-	2/4/4/4	-
2	GOL	A	306	-	-	2/4/4/4	-
2	GOL	A	307	-	-	2/4/4/4	-
2	GOL	A	308	-	-	2/4/4/4	-
3	PEG	A	309	-	-	1/4/4/4	-
2	GOL	B	301	-	-	0/4/4/4	-
3	PEG	B	302	-	-	2/4/4/4	-
2	GOL	C	301	-	-	0/4/4/4	-
2	GOL	C	302	-	-	2/4/4/4	-
2	GOL	C	303	-	-	2/4/4/4	-
2	GOL	C	304	-	-	2/4/4/4	-
2	GOL	C	305	-	-	2/4/4/4	-
2	GOL	C	306	-	-	2/4/4/4	-
2	GOL	C	307	-	-	2/4/4/4	-
3	PEG	C	308	-	-	0/4/4/4	-
5	TRS	D	501	-	-	0/9/9/9	-
5	TRS	D	502	-	-	0/9/9/9	-
2	GOL	D	503	-	-	2/4/4/4	-
2	GOL	D	504	-	-	2/4/4/4	-
2	GOL	D	505	-	-	2/4/4/4	-
3	PEG	D	506	-	-	1/4/4/4	-

There are no bond length outliers.

There are no bond angle outliers.

There are no chirality outliers.

All (38) torsion outliers are listed below:

Mol	Chain	Res	Type	Atoms
2	A	305	GOL	O1-C1-C2-C3
2	A	306	GOL	O1-C1-C2-C3
2	A	301	GOL	O1-C1-C2-C3
2	C	304	GOL	O1-C1-C2-C3
2	A	304	GOL	O1-C1-C2-C3
2	C	306	GOL	O1-C1-C2-C3

Continued on next page...

CONFIDENTIAL VALIDATION REPORT

Continued from previous page...

Mol	Chain	Res	Type	Atoms
2	A	302	GOL	O1-C1-C2-C3
2	D	503	GOL	O1-C1-C2-C3
2	C	307	GOL	O1-C1-C2-C3
2	D	505	GOL	O1-C1-C2-O2
2	A	307	GOL	O2-C2-C3-O3
2	A	303	GOL	O1-C1-C2-O2
2	A	303	GOL	O1-C1-C2-C3
2	C	302	GOL	O1-C1-C2-O2
2	A	308	GOL	O1-C1-C2-C3
2	C	303	GOL	O1-C1-C2-C3
2	D	504	GOL	O1-C1-C2-C3
2	C	302	GOL	O1-C1-C2-C3
2	C	305	GOL	O1-C1-C2-C3
2	D	505	GOL	O1-C1-C2-C3
2	A	307	GOL	C1-C2-C3-O3
2	A	308	GOL	O1-C1-C2-O2
2	A	306	GOL	O1-C1-C2-O2
2	A	301	GOL	O1-C1-C2-O2
2	C	304	GOL	O1-C1-C2-O2
2	A	302	GOL	O1-C1-C2-O2
2	C	305	GOL	O1-C1-C2-O2
2	C	307	GOL	O1-C1-C2-O2
2	A	305	GOL	O1-C1-C2-O2
2	A	304	GOL	O1-C1-C2-O2
2	C	306	GOL	O1-C1-C2-O2
3	B	302	PEG	O1-C1-C2-O2
2	D	503	GOL	O1-C1-C2-O2
3	B	302	PEG	C1-C2-O2-C3
3	D	506	PEG	C1-C2-O2-C3
2	D	504	GOL	O1-C1-C2-O2
2	C	303	GOL	O1-C1-C2-O2
3	A	309	PEG	C4-C3-O2-C2

There are no ring outliers.

6 monomers are involved in 7 short contacts:

Mol	Chain	Res	Type	Clashes	Symm-Clashes
2	A	301	GOL	1	0
3	A	309	PEG	1	0
2	B	301	GOL	1	0
3	B	302	PEG	1	0
2	C	301	GOL	1	0

Continued on next page...

CONFIDENTIAL VALIDATION REPORT

Continued from previous page...

Mol	Chain	Res	Type	Clashes	Symm-Clashes
2	D	504	GOL	2	0

5.7 Other polymers (i)

There are no such residues in this entry.

5.8 Polymer linkage issues (i)

There are no chain breaks in this entry.

CONFIDENTIAL VALIDATION REPORT

6 Fit of model and data (i)

6.1 Protein, DNA and RNA chains (i)

In the following table, the column labelled '#RSRZ> 2' contains the number (and percentage) of RSRZ outliers, followed by percent RSRZ outliers for the chain as percentile scores relative to all X-ray entries and entries of similar resolution. The OWAB column contains the minimum, median, 95th percentile and maximum values of the occupancy-weighted average B-factor per residue. The column labelled 'Q< 0.9' lists the number of (and percentage) of residues with an average occupancy less than 0.9.

Mol	Chain	Analysed	<RSRZ>	#RSRZ>2			OWAB(Å^2)	Q<0.9
1	A	227/271 (83%)	-0.47	0	100	100	19, 30, 52, 64	0
1	B	228/271 (84%)	-0.45	2 (0%)	84	83	18, 32, 59, 76	0
1	C	227/271 (83%)	-0.45	1 (0%)	92	91	20, 31, 52, 70	0
1	D	229/271 (84%)	-0.50	3 (1%)	77	75	18, 30, 58, 72	0
All	All	911/1084 (84%)	-0.47	6 (0%)	87	86	18, 31, 56, 76	0

All (6) RSRZ outliers are listed below:

Mol	Chain	Res	Type	RSRZ
1	B	75	ALA	4.0
1	D	75	ALA	3.4
1	B	77	TYR	2.7
1	D	77	TYR	2.2
1	D	76	GLU	2.2
1	C	77	TYR	2.1

6.2 Non-standard residues in protein, DNA, RNA chains (i)

There are no non-standard protein/DNA/RNA residues in this entry.

6.3 Carbohydrates (i)

There are no carbohydrates in this entry.

6.4 Ligands (i)

In the following table, the Atoms column lists the number of modelled atoms in the group and the number defined in the chemical component dictionary. The B-factors column lists the minimum,

CONFIDENTIAL VALIDATION REPORT

median, 95th percentile and maximum values of B factors of atoms in the group. The column labelled 'Q< 0.9' lists the number of atoms with occupancy less than 0.9.

Mol	Type	Chain	Res	Atoms	RSCC	RSR	B-factors(Å^2)	Q<0.9
5	TRS	D	502	8/8	0.68	0.24	40,56,72,80	0
2	GOL	C	302	6/6	0.70	0.19	43,55,60,63	0
2	GOL	D	505	6/6	0.70	0.25	51,55,61,63	0
2	GOL	D	504	6/6	0.72	0.20	40,44,52,52	0
2	GOL	A	308	6/6	0.75	0.34	53,59,68,74	0
3	PEG	B	302	7/7	0.75	0.20	46,51,57,59	0
2	GOL	C	304	6/6	0.75	0.19	43,49,56,57	0
2	GOL	C	303	6/6	0.75	0.29	60,66,68,74	0
4	K	A	311	1/1	0.75	0.09	54,54,54,54	0
2	GOL	A	307	6/6	0.76	0.29	31,48,51,52	0
2	GOL	A	303	6/6	0.76	0.21	49,52,55,59	0
2	GOL	C	301	6/6	0.79	0.27	42,45,58,63	0
2	GOL	A	302	6/6	0.83	0.16	39,48,59,59	0
2	GOL	C	305	6/6	0.83	0.16	42,46,55,58	0
4	K	D	507	1/1	0.84	0.10	52,52,52,52	0
2	GOL	A	301	6/6	0.85	0.18	41,52,57,60	0
2	GOL	A	304	6/6	0.85	0.17	45,48,51,55	0
3	PEG	C	308	7/7	0.85	0.15	40,45,52,56	0
4	K	B	304	1/1	0.86	0.08	47,47,47,47	0
3	PEG	D	506	7/7	0.86	0.18	44,45,55,57	0
3	PEG	A	309	7/7	0.88	0.15	38,43,48,53	0
4	K	C	310	1/1	0.88	0.13	56,56,56,56	0
2	GOL	C	307	6/6	0.90	0.15	32,37,42,42	0
4	K	C	309	1/1	0.90	0.07	42,42,42,42	0
2	GOL	A	305	6/6	0.91	0.17	38,41,46,51	0
2	GOL	D	503	6/6	0.91	0.17	32,42,45,60	0
5	TRS	D	501	8/8	0.91	0.22	37,47,53,55	0
2	GOL	C	306	6/6	0.94	0.11	30,35,44,46	0
2	GOL	A	306	6/6	0.94	0.13	29,33,37,47	0
4	K	A	310	1/1	0.94	0.06	41,41,41,41	0
2	GOL	B	301	6/6	0.96	0.12	28,34,39,48	0
4	K	D	508	1/1	0.97	0.07	39,39,39,39	0
4	K	B	303	1/1	0.98	0.05	39,39,39,39	0

6.5 Other polymers (i)

There are no such residues in this entry.

CONFIDENTIAL VALIDATION REPORT
