# Supplementary material for: Direct control of CAR T cells through small molecule-regulated antibodies
Source: Nat Commun. 2021 Jan 29;12:710. doi: 10.1038/s41467-020-20671-6 (PMC7846603; doi:10.1038/s41467-020-20671-6)
Supplement: Supplementary file 3 — Reporting Summary [file 41467_2020_20671_MOESM3_ESM.pdf]

nature research

Corresponding author(s): Thomas Van Blarcom

Last updated by author(s): Nov 13, 2020

Reporting Summary

Nature Research wishes to improve the reproducibility of the work that we publish. This form provides structure for consistency and transparency in reporting. For further information on Nature Research policies, see our Editorial Policies and the Editorial Policy Checklist.

Statistics

For all statistical analyses, confirm that the following items are present in the figure legend, table legend, main text, or Methods section.

n/a	Confirmed	
☐	☒	The exact sample size (*n*) for each experimental group/condition, given as a discrete number and unit of measurement
☐	☒	A statement on whether measurements were taken from distinct samples or whether the same sample was measured repeatedly
☐	☒	The statistical test(s) used AND whether they are one- or two-sided<br>*Only common tests should be described solely by name; describe more complex techniques in the Methods section.*
☒	☐	A description of all covariates tested
☒	☐	A description of any assumptions or corrections, such as tests of normality and adjustment for multiple comparisons
☐	☒	A full description of the statistical parameters including central tendency (e.g. means) or other basic estimates (e.g. regression coefficient) AND variation (e.g. standard deviation) or associated estimates of uncertainty (e.g. confidence intervals)
☐	☒	For null hypothesis testing, the test statistic (e.g. *F*, *t*, *r*) with confidence intervals, effect sizes, degrees of freedom and *P* value noted<br>*Give P values as exact values whenever suitable.*
☒	☐	For Bayesian analysis, information on the choice of priors and Markov chain Monte Carlo settings
☒	☐	For hierarchical and complex designs, identification of the appropriate level for tests and full reporting of outcomes
☒	☐	Estimates of effect sizes (e.g. Cohen's *d*, Pearson's *r*), indicating how they were calculated

Our web collection on statistics for biologists contains articles on many of the points above.

Software and code

Policy information about availability of computer code

Data collection	MSD Discovery Workbench 4.0, BD FACSDiva v8.0
Data analysis	The data were analyzed using Microsoft Excel 2016, Biacore SK Evaluation Software vl.1.1.7442, Biacore 4000 Evaluation Software vl.1, PHENIX, MolProbity, Pymol, COOT, XDS, FlowJo software v10, Living Image, Graphpad Prism v6.0.

For manuscripts utilizing custom algorithms or software that are central to the research but not yet described in published literature, software must be made available to editors and reviewers. We strongly encourage code deposition in a community repository (e.g. GitHub). See the Nature Research guidelines for submitting code & software for further information.

Data

Policy information about availability of data

All manuscripts must include a data availability statement. This statement should provide the following information, where applicable:

- Accession codes, unique identifiers, or web links for publicly available datasets
- A list of figures that have associated raw data
- A description of any restrictions on data availability

The atomic coordinates and structure factors have been deposited in the Protein Data Bank, www.wwpdb.org (PDB codes 6UUP and 6UY3). Source data supporting the graphs and charts presented in the text are provided with this paper. The authors declare that all other data supporting the findings of this study are available within the paper and its supplementary information files.

Field-specific reporting

Please select the one below that is the best fit for your research. If you are not sure, read the appropriate sections before making your selection.

- [x] Life sciences
- [ ] Behavioural & social sciences
- [ ] Ecological, evolutionary & environmental sciences

For a reference copy of the document with all sections, see nature.com/documents/nr-reporting-summary-flat.pdf

Life sciences study design

All studies must disclose on these points even when the disclosure is negative.

Sample size	No statistical methods were used to determine sample size. For calculating statistics in in vitro studies, a minimum of triplicates was chosen to allow for this. For animal studies, 5 mice were included for each treatment group based on other published studies for establishing proof of concept.
Data exclusions	No data were excluded.
Replication	The replication number and the number of donors tested are indicated in the legend of corresponding figures, where applicable. All attempts at replication were successful. The in vivo study shown was performed once but every effort was made to include critical controls in the study (e.g. untransduced T cell cohort and vehicle treatment cohort).
Randomization	Animals were randomly divided into experimental groups in such a way that prior to T cell injection, mice in each cohort carried similar average tumor burden. For in vitro experiments, sample were allocated to identical cell-culture wells and there is no reason to believe the spatial location of the sample/well influenced experimental results.
Blinding	Investigators were not blinded. For animal studies, mice in control and test groups were handled in a consistent manner to prevent study bias, and only quantitative measures were used to make conclusions. Fully blinded experiments were not possible due to personnel availability to accommodate such situations.

Reporting for specific materials, systems and methods

We require information from authors about some types of materials, experimental systems and methods used in many studies. Here, indicate whether each material, system or method listed is relevant to your study. If you are not sure if a list item applies to your research, read the appropriate section before selecting a response.

Materials & experimental systems

n/a	Involved in the study	
[ ]	[x]	Antibodies
[ ]	[x]	Eukaryotic cell lines
[x]	[ ]	Palaeontology and archaeology
[ ]	[x]	Animals and other organisms
[ ]	[x]	Human research participants
[x]	[ ]	Clinical data
[x]	[ ]	Dual use research of concern

Methods

n/a	Involved in the study	
[x]	[ ]	ChIP-seq
[ ]	[x]	Flow cytometry
[x]	[ ]	MRI-based neuroimaging

Antibodies

Antibodies used	His-tag antibody (R&D Systems #MAB050), anti-human Fc polyclonal antibody (Southern Biotech #2014-01), anti-human CD33-PE (Biolegend #366608), anti-human CD3-BV421 (Biolegend #617344), anti-human CD69-BV605 (Biolegend #310938), anti-mouse CD45-BV421 (Biolegend #103134), anti-human CD62L-BV605 (Biolegend #304834), anti-human CD137-PE/Cy7 (Biolegend #309818), anti-human CD3-APC (Biolegend #317318), anti-V5-FITC (Invitrogen #MA1-80281)
Validation	All Biolegend antibodies were validated by the supplier and used according to the profile of manufacturer.<br>Anti-human CD33-PE (clone P67.6) was validated here: https://www.biolegend.com/en-us/products/pe-anti-human-cd33-antibody-12158<br>Anti-human CD3-BV421 (clone OKT3) was validated here: https://www.biolegend.com/en-us/products/brilliant-violet-421-anti-human-cd3-antibody-11976<br>Anti-human CD69-BV605 (clone FN50) was validated here: https://www.biolegend.com/en-us/products/brilliant-violet-605-anti-human-cd69-antibody-8704<br>Anti-mouse CD45-BV421 (clone 30-F11) was validated here: https://www.biolegend.com/en-us/products/brilliant-violet-421-anti-mouse-cd45-antibody-7253<br>Anti-human CD62L-BV605 (clone DREG-56) was validated here: https://www.biolegend.com/en-us/products/brilliant-violet-605-anti-human-cd62l-antibody-8554<br>Anti-human CD137-PE/Cy7 (clone 4B4-1) was validated here: https://www.biolegend.com/en-us/products/pe-cyanine7-anti-human-

cd137-4-1bb-antibody-6540
Anti-human CD3-APC (clone OKT3) was validated here: https://www.biolegend.com/en-us/products/apc-anti-human-cd3-antibody-6198
Anti-V5-FITC (clone SV5-Pk1) specific staining was compared to FMO samples.
Anti-His antibody (clone AD1.1.10) was validated here: https://www.rndsystems.com/products/his-tag-antibody-ad1110_mab050
Anti-human Fc antibody (polyclonal) was validated here: https://www.southernbiotech.com/?catno=2014-01&type=Polyclonal#&panel2-1

Eukaryotic cell lines

Policy information about cell lines

Cell line source(s)	Expi293 (Thermo Fisher Scientific), HEK293T (ATCC), MV4-11 (ATCC), K562 (ATCC)
Authentication	All cell lines authenticated by STR method.
Mycoplasma contamination	All cell lines were tested for mycoplasma contamination and they were free of mycoplasma contamination.
Commonly misidentified lines (See ICLAC register)	None of the used cell lines is listed in ICLAC database.

Animals and other organisms

Policy information about studies involving animals; ARRIVE guidelines recommended for reporting animal research

Laboratory animals	In this study were used 6-8 week old female NSG mice (NOD.Cg-Prkdcscid IL-2R!tm1Wjl/SzJ) obtained from The Jackson Laboratory. The mice were housed in a pathogen-free BSL2 biohazard facility with unrestricted access to water and food. The ambient temperature was restricted to 65 to 75 degrees F with 40-60% humidity. Mice were exposed to a 12:12h light-dark cycle.
Wild animals	This study did not involve wild animals
Field-collected samples	This study did not involve field-collected samples
Ethics oversight	All procedures were performed in accordance with regulations and established guidelines and were reviewed and approved by Pfizer's Institutional Animal Care and Use Committee.

Note that full information on the approval of the study protocol must also be provided in the manuscript.

Human research participants

Policy information about studies involving human research participants

Population characteristics	Anonymous healthy human donors (male and female age 30-50)
Recruitment	Human PBMCs were collected from anonymous healthy human donors through Standord Blood Bank.
Ethics oversight	Studies were carried out in accordance with Prizer IRB/IEC policies.

Note that full information on the approval of the study protocol must also be provided in the manuscript.

Flow Cytometry

Plots

Confirm that:

- [x] The axis labels state the marker and fluorochrome used (e.g. CD4-FITC).
- [x] The axis scales are clearly visible. Include numbers along axes only for bottom left plot of group (a 'group' is an analysis of identical markers).
- [x] All plots are contour plots with outliers or pseudocolor plots.
- [x] A numerical value for number of cells or percentage (with statistics) is provided.

Methodology

Sample preparation	Human peripheral blood was obtained from anonymous healthy donors through Stanford Blood Bank and was used in accordance with Pfizer IRB/IEC policies. Peripheral blood leukocytes were isolated with Ficoll-Paque PLUS according to the manufacturer's protocol. Pan T cells were isolated via negative selection using Pan T Cell Isolation Kit, human (Miltenyi Biotec) and cryopreserved in 90% human AB serum (Gemini Bio-Products) and 10% DMSO.<br>~100,000 T cells were harvested, pelleted (300xg, 5min), and incubated in 100 microliters of staining buffer with indicated antibodies for 30 minutes at room temperature in the dark. Cells were then washed 2x with staining buffer, resuspended in 200 microliters before data collection.<br>Cell cultures (~100,000 cells per sample) were harvested, pelleted (300g, 5min), and incubated in 100 microliters of staining

	buffer with indicated antibodies for 30 minutes at room temperature in the dark. Cells were then washed 2x with staining buffer, resuspended in 200 microliters before data collection.<br>Samples were analyzed by flow cytometry using BD FACSDiva (BD Biosciences), at least 10000 events were collected per sample. Collected data were processed by FlowJo (Tree Star).
Instrument	BD LSRII (BD Biosciences)
Software	Data was collected using BD FACSDiva. Data was analyzed using FlowJo v10
Cell population abundance	Cell sorting not employed
Gating strategy	Using the FSC-A/SSC-A gating, debris was removed by gating on the main cell population. Using the FSC-H/FSC-A, single cell population was selected. Multicolor samples were compensated with the help of single-color controls. Further gating strategies depend on the particular experimental setup. Identical gating strategy was applied to all samples within each experiment.

☒ Tick this box to confirm that a figure exemplifying the gating strategy is provided in the Supplementary Information.
